# Beyond Artists’ Colors: A Spectral Reference Database for the Identification of β-Naphthol and Triarylcarbonium Colorants by MeV SIMS

**DOI:** 10.1021/acsomega.4c03634

**Published:** 2024-09-11

**Authors:** Teodora Raicu, Matea Krmpotić, Zdravko Siketić, Iva Bogdanović Radović, Katja Sterflinger, Dubravka Jembrih-Simbürger

**Affiliations:** † Institute for Natural Sciences and Technology in the Arts, Academy of Fine Arts Vienna, Augasse 2-6, Vienna A-1090, Austria; ‡ Division of Experimental Physics, Laboratory for Ion Beam Interactions, Ruđer Bošković Institute, Bijenička cesta 54, Zagreb HR-10000, Croatia

## Abstract

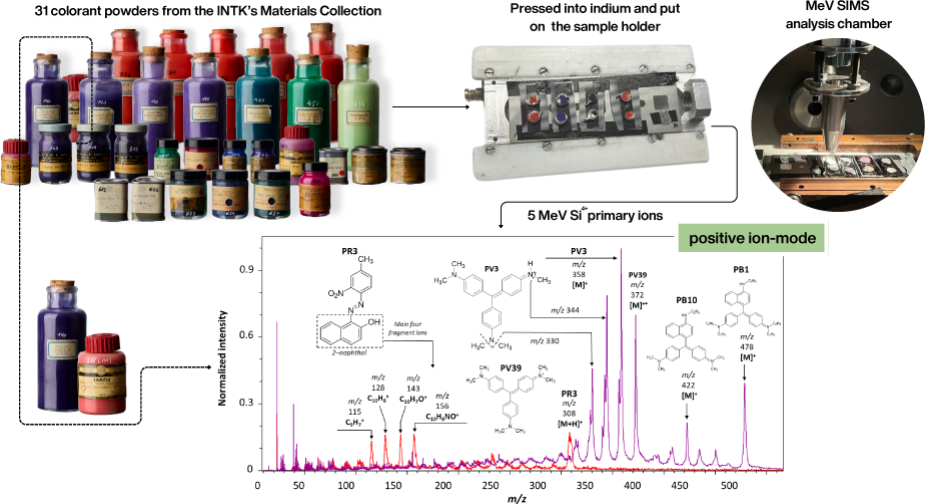

β-Naphthol and triarylcarbonium colorants were often used by modern and contemporary artists in materials such as inks and paints. Their poor stability and ability to fade upon exposure to light make their identification in artworks crucial for planning exhibitions and preventive conservation. Secondary ion mass spectrometry with MeV primary ions (MeV SIMS) is necessary when analyzing synthetic organic colorants (SOCs) with similar molecular structures due to its advantages in high sensitivity and soft ionization, which causes a low fragmentation of organic molecules. In this work, we applied MeV SIMS with 5 MeV Si^4+^ to identify selected β-naphthol and triarylcarbonium colorants from the 19th/20th century Materials Collection kept at the Academy of Fine Arts Vienna. The collection contains SOCs from renowned companies, such as I.G. Farben and I.C.I., and serves as a unique source of reference materials in the analysis of artworks. Previous research on these colorants with X-ray fluorescence analysis (XRF), micro-Raman, and Fourier transform infrared (FTIR) spectroscopies failed in the case of colorant mixtures. Similar spectral features of the SOCs within one chemical class and their low concentrations in mixtures did not lead to their identification using these techniques. MeV SIMS detected molecular ions or protonated molecules in the positive-ion mode. In the negative-ion mode, the functional groups (NO_2_^–^ and SO_3_^–^) of β-naphthol lakes/pigments and heteropolyacid species (WO_3_^–^ and MoO_3_^–^) characteristic of triarylcarbonium toners were determined. The results demonstrate that MeV SIMS is highly effective for identifying β-naphthol and triarylcarbonium colorants in mixtures and distinguishing between pigments, toners, lakes, and dyes.

## Introduction

Synthetic organic colorants (SOCs) have imparted color to various industrial products since Sir William Perkin synthesized mauveine, the first artificial dye, in 1856. Today, over 4000 colorants are frequently employed,^1^ and some of the most prevalent are β-naphthol and triarylcarbonium pigments, lakes (obtained by complexation of pigments), and toners (obtained by precipitation of dyes), as well as azo and triarylmethane dyes.^2,3^ These SOCs are found in industrial products such as writing and printing inks, plastics, paints, cosmetics, and textiles,^4^ which were also included in the works of modern and contemporary artists. Mark Rothko^5^ and Pablo Picasso^6^ used β-naphthol pigments and lakes, and Vincent van Gogh^7^ and Lucio Fontana^8^ used triarylmethane dyes and possibly triarylcarbonium toners. Since prolonged exposure to light causes these SOCs to fade, their identification in artworks is essential for establishing a preventive conservation plan that safeguards cultural heritage objects during display and storage.

Based on their chemical manufacturing, the colorants from the β-naphthol class are classified as either pigments or lakes, while the colorants from the triarylcarbonium class as toners.^9^ In the cultural heritage field, β-naphthol pigments are known as azo pigments due to the diazonium salts used in coupling reactions with phenols.^10^ They consist of 2-naphthol (or β-naphthol) as the coupling component and lack acid or basic groups.^11^ Conversely, β-naphthol lakes, which include a sulfonic acid group, are formed through complexation with, e.g., Ca^2+^, Ba^2+^, and Sr^2+^.^12,13^ They resist migration and solvents better than the pigments from the β-naphthol class, but they have lower lightfastness.^12^ Triarylcarbonium toners have been used since 1913^14^ when a novel method was discovered for precipitating triarylmethane dyes. The production of these toners was likely motivated by their superior lightfastness in comparison to that of triarylmethane dyes.^11,15^ The precipitation involved replacing the chlorine anion (specific to dyes) with heteropolyacids such as phosphotungstic acid (PTA), phosphomolybdic acid (PMA), phosphotungstomolybdic acid (PTMA, a mixture of PTA and PMA), silicomolybdic acid (SMA), and copper ferrocyanide (CF).^11,12,16,17^ As a result, the only difference in chemical composition between triarylcarbonium toners and triarylmethane dyes is the presence of chlorine (characteristic of dyes) or heteropolyacids (characteristic of toners).

Due to the high artistic and historical value of cultural heritage objects, only small (often <100 μg) and unique samples are generally analyzed, if at all. Consequently, the identification of β-naphthol and triarylcarbonium colorants in such samples is challenging because (1) they are frequently intermixed to achieve the desired colors and (2) they are present in different concentrations depending on their tinctorial strength and other properties. Despite these challenges, researchers in the fields of cultural heritage and forensic science attempted to identify these SOCs using various analytical techniques. These were noninvasive and nondestructive techniques such as X-ray fluorescence (XRF) and particle-induced X-ray emission (PIXE),^14,18,19^ spectrofluorimetry,^20^ Raman spectroscopy,^21,22^ as well as minimally invasive and nondestructive techniques such as Fourier transform infrared spectroscopy (FTIR),^23,24^ and secondary ion mass spectrometry with primary ions in the keV range (keV SIMS).^25,26^ Additionally, invasive and destructive techniques were used, such as pyrolysis gas chromatography mass spectrometry (Py-GC/MS),^27^ direct temperature-resolved mass spectrometry (DTMS),^24^ matrix-assisted laser desorption/ionization mass spectrometry (MALDI-MS),^28^ and high-performance liquid chromatography mass spectrometry (HPLC-MS).^29^

According to the literature, XRF and PIXE highlighted the presence of metals specific to β-naphthol lakes, such as Ca,^19^ and the heteropolyacid characteristic of triarylcarbonium toners, such as W and Mo.^14,18^ Spectrofluorimetry detected unmixed β-naphthol pigments in oil-paint mockups.^20^ Raman spectroscopy identified β-naphthol lakes in artist materials^21,22^ and samples taken from artworks,^30^ although it was unsuitable for identifying triarylcarbonium toners due to fluorescence. Nonetheless, such toners were found in two Talens paints,^21^ but challenges arose in identifying all constituent SOCs within colorant mixtures. In contrast, FTIR provided only the chemical class,^24^ as overlapping bands hindered the differentiation between compounds within colorant mixtures.^31^ Therefore, common noninvasive analytical techniques often fail to draw clear distinctions between these colorant classes. Due to its high fragmentation, Py-GC/MS yielded no molecular ions or large fragment ions. Rather, low-mass compounds were detected, such as 2-naphthol (*m*/*z* 144) in β-naphthol pigments and lakes and benzenamines in triarylcarbonium toners (*m*/*z* 134–254).^27,32^ On the other hand, DTMS showed molecular and large fragment ions of SOCs, ensuring the identification of such colorants.^24^ Similarly, MALDI-MS and HPLC-MS successfully identified triarylmethane dyes and triarylcarbonium toners through the observation of molecular ions.^28,29^ However, both techniques require chemical treatment and sample consumption, which are infeasible when dealing with small and valuable samples.

Forensic science employs SIMS with keV primary ions (keV SIMS) to discriminate ballpoint pen inks that contain colorants like triarylmethane dyes.^25,26^ However, this method significantly fragments organic molecules, potentially missing some colorants in mixtures due to unclear identification or low intensity of molecular ions or (de)protonated molecules. To address this, forensic scientists use thin-layer chromatography (TLC) before keV SIMS analysis to separate the constituent dyes in the inks and improve analytical discrimination.^25^ Nevertheless, this technique is unsuitable for heritage science applications, as it involves the use of solvents on very small ink samples for component separation, resulting in sample loss. In contrast, MeV SIMS is an accelerator-based technique that provides more than 10^3^ times higher yield than keV SIMS,^33^ with no chemical treatment required and no sample loss. It effectively identifies molecular ions and/or (de)protonated molecules of synthetic organic pigments, even in mixtures.^33,34,35^ The main difference between keV SIMS and MeV SIMS lies in the interaction of primary ions with the sample surface: in keV SIMS, ion sputtering through nuclear stopping dominates, while in MeV SIMS, electronic stopping regime prevails, allowing softer desorption and, thus, higher yields of molecular ions and reduced fragmentation.^36^

The Institute for Natural Sciences and Technology in the Arts (INTK) at the Academy of Fine Arts Vienna has a unique materials collection dated to 19th/20th century, including over 1,000 synthetic organic colorants (SOCs) stored as powders in original or repurposed containers. These materials are used as references for identifying colorants and studying the degradation phenomena in cultural heritage objects. Approximately 10% of these SOCs contain β-naphthol and triarylcarbonium pigments, lakes, and toners, which were manufactured or resold by renowned companies: I.C.I. (UK), G. Siegle & Co. (Germany), J.W. & T.A. Smith (UK), I.G. Farben (Germany), Geigy (Switzerland), J.S. & W.R. Eakins (USA), Kast + Ehinger (Germany), Cappelle Frères (France), and Bayer (Germany). The historical information available on these SOCs was summarized by Schäning.^37^ It is known that, e.g., G. Siegle & Co. prepared and sold mixtures of various colorants.^37^ However, it is not mandatory for manufacturers or resellers to disclose the complete list of colorants present in their products. Still, when such materials are used as references, their composition must be known. In previous work, common analytical techniques, such as XRF, FTIR,^31,37^ and micro-Raman, were applied for the characterization of β-naphthol and triarylcarbonium colorants from the INTK collection. For colorant mixtures, no clear results were obtained. Micro-Raman mostly identified only one SOC in mixtures, while the presence of heteropolyacids was deducible by XRF because of the detection of W and/or Mo, which indicated the presence of PTA, PMA, or PTMA. FTIR failed to characterize mixtures due to the multitude of bands in the spectra that were difficult to be attributed to similar molecular structures.^31,37^

In this study, we used MeV SIMS with 5 MeV Si^4+^ primary ions to identify colorants and colorant mixtures from the INTK Materials Collection, specifically from the β-naphthol and triarylcarbonium classes. We also aimed to differentiate among pigments, lakes, toners, and dyes. The obtained MeV SIMS spectra will enrich the open access INTK pigment database (currently under construction). The database includes historical information about colorant samples, XRF, FTIR, Raman results, images of individual colorants in their respective containers, color index names and numbers, etc.

## Experimental Section

### Samples and Sample Preparation

Thirty-one colorant samples were analyzed, six β-naphthol and twenty-five triarylcarbonium colorants, which are summarized in Table 1. The colorants present in each sample are listed according to the internal INTK’s material database and the previous multianalytical approach (XRF, FTIR,^31,37^ and micro-Raman). A more detailed evaluation of these former results is presented in Table S3.

**Table 1:** Colorants from the INTK’s 19th/20th Century Materials Collection Analyzed with MeV SIMS^a^

inv. no. = sample	chemical class	bottle name	manufacturer/reseller	color	color index name	chemical formula	monoisotopic mass (Da)
140	triarylcarbonium	Violett 62 492 N	G. Siegle & Co.	violet	PV3	C_24_H_28_N_3_	358.2278
141	triarylcarbonium	Fanalviolett R Supra	I.G. Farben	violet	PV3	C_24_H_28_N_3_	358.2278
142	triarylcarbonium	Rotviolett D 447	G. Siegle & Co.	violet	PV3	C_24_H_28_N_3_	358.2278
144	triarylcarbonium	Blauviolett D 447	G. Siegle & Co.	violet	PV3	C_24_H_28_N_3_	358.2278
215	β-naphthol	Monolite Fast Scarlet CAS Granulates	I.C.I.	orange	PR3	C_17_H_13_N_3_O_3_	307.0957
226	β-naphthol	Echtrot 1	Siegle	red	PR4	C_16_H_10_ClN_3_O_3_	327.0411
231	β-naphthol	Helioechtrot RL	Bayer	red	PR3	C_17_H_13_N_3_O_3_	307.0957
232	β-naphthol, Na salt	Lithol Red R 4593	Unknown	red	PR49	C_20_H_13_N_2_O_4_S (without Na)	377.0596
248	β-naphthol, Ba salt	Spektralrot gelbl. Extr.	Kast + Ehinger	red	PR53:1	C_17_H_12_ClN_2_O_4_S (without Ba)	375.0212
250	β-naphthol	Litholechtscharlach RN	I.G. Farben	red	PR3	C_17_H_13_N_3_O_3_	307.0957
411	triarylcarbonium	Fanalgrün	I.G. Farben	green	PG1	C_27_H_34_N_2_	386.2722
450	triarylcarbonium	Spektraltiefgrün gelbl. 2320	Kast + Ehinger	green	PG1	C_27_H_34_N_2_	386.2722
					PY18	C_17_H_19_N_2_S	283.1263
					PG4	C_23_H_26_N_2_	330.2096
464	triarylcarbonium	Sieglegrün D451	G. Siegle & Co.	green	PG1	C_27_H_34_N_2_	386.2722
					PY18	C_17_H_19_N_2_S	283.1263
					PG4	C_23_H_26_N_2_	330.2096
489	triarylcarbonium	Fastel Pink B Powder	I.C.I.	red	PR81	C_28_H_31_N_2_O_3_	443.2329
503	triarylcarbonium	Dragon Purple	J.S. & W.R. Eakins	violet	PV3	C_24_H_28_N_3_	358.2278
504	triarylcarbonium	Climatone Blue Toner	J.S. & W.R. Eakins	blue-violet	PB1	C_33_H_40_N_3_	478.3217
513	triarylcarbonium	Climatone Purple Toner	J.S. & W.R. Eakins	violet	PV3	C_24_H_28_N_3_	358.2278
537	triarylcarbonium	Fastel Yellow Green GA Supra Powder	I.C.I.	green	PG1	C_27_H_34_N_2_	386.2722
					PY18	C_17_H_19_N_2_S	283.1263
546	triarylcarbonium	Brillfast Red 6114	J.W. & T.A. Smith	red	PR81	C_28_H_31_N_2_O_3_	443.2329
556	triarylcarbonium	Fastel Blue B Supra Powder	I.C.I.	blue	PB1	C_33_H_40_N_3_	478.3217
573	triarylcarbonium	Fastel Violet R Supra Powder	I.C.I.	violet	PV3	C_24_H_28_N_3_	358.2278
577	triarylcarbonium	Fastel Pink 2B Supra Powder	I.C.I.	red	PR81	C_28_H_31_N_2_O_3_	443.2329
584	triarylcarbonium	Irgalite azur blue TCR	Geigy	blue	PB3	C_25_H_28_ClN_2_	391.2941
594	triarylcarbonium	Vert Clair Lumière	Cappelle Frères	green	PG1	C_27_H_34_N_2_	386.2722
					PY18	C_17_H_19_N_2_S	283.1263
					PG4	C_23_H_26_N_2_	330.2096
595	triarylcarbonium	Vert forte Lumière	Cappelle Frères	green	PG1	C_27_H_34_N_2_	386.2722
602	triarylcarbonium	Irgalite Blue TCS	Geigy	blue	PB3	C_25_H_28_ClN_2_	391.2941
606	triarylcarbonium	Irgalite Violet TCR	Geigy	violet	PV3	C_24_H_28_N_3_	358.2278
623	triarylcarbonium	Brillfast Sky Blue 3862	J.W. & T.A. Smith	blue	PB3	C_25_H_28_ClN_2_	391.2941
624	triarylcarbonium	Brillfast Maltese Blue 3591	J.W. & T.A. Smith	blue	PB1	C_33_H_40_N_3_	478.3217
627	triarylcarbonium	Brillfast Deep Green	J.W. & T.A. Smith	green	PG1	C_27_H_34_N_2_	386.2722
867	triarylcarbonium	Fastel Pink R Supra Powder	I.C.I.	red	PR81	C_28_H_31_N_2_O_3_	443.2329

a The details regarding the constituent colorants were taken from the former XRF and FTIR results^37^ and micro-Raman (unpublished data)

For the MeV SIMS analysis, a small amount of colorant powder was pressed onto the flat surface of indium drops (99.99%, tear drops, *Alpha Aesar*, USA) by using a small aluminum plate. Indium was selected as the sample support material because previous studies have proven its superiority in extracting secondary molecular ions over other supports, such as carbon tape.^33^

### Experimental Procedure

Samples were analyzed using the MeV SIMS setup with a linear TOF spectrometer at the heavy ion microbeam beamline at the Ruđer Bošković Institute (RBI).^38^ This setup has already been employed and is described in our work concerning the implementation of MeV SIMS for identifying synthetic organic pigments present in artworks.^33−34^ A pulsed 5 MeV Si^4+^ primary ion beam was focused to a 10 × 10 μm^2^ and scanned up to 1000 × 1000 μm^2^ sample area. The beam fluence was kept below the static limit of 1 × 10^12^ primary ions/cm^2^ for all measurements. Measurements were performed in a high vacuum environment, between 10^–6^ and 10^–7^ mbar, in the positive- and negative-ion modes, with target potentials set at +5 and −4 kV, respectively. The SPECTOR software package^38^ was used for the control of experimental parameters, data acquisition, and mass spectra calibration.

### Data Processing and Evaluation

Mass calibration was performed for each spectrum to account for differences in the sample surface geometry, which are known to affect the extraction and arrival time of secondary ions in SIMS techniques.^39^ Unfortunately, there were no MeV SIMS reference spectra for these colorant classes that could guide the calibration. Thus, ions were selected from both the lower and higher mass regions, which were expected to be identified in the mass spectra. In the lower mass region, we used H^+^, H_2_^+^, C_*x*_H_*y*_^+^, and other inorganic species such as NaOH^+^ and BaCl^+^ in the positive-ion mode and H^–^, CN^–^, CNO^–^, PO_3_^–^, SO_3_^–^, and NO_3_^–^ in the negative-ion mode. In the higher mass region of the measured spectra (*m*/*z* from 250 to 500), the molecular ions/(de)protonated molecules of the analyzed SOCs assisted in the calibration procedure. Their masses and main fragmentation patterns were drawn from the literature focused on the analysis of these colorants using other MS techniques, such as DTMS,^24^ MALDI-MS,^28,40^ and/or TOF-MS performed with primary ions in the keV range.^41^ Additionally, the mass spectra from *The Static SIMS Library* (version 4, SurfaceSpectra Ltd.) were used for matching some of the compounds present in the analyzed colorants. ChemDraw (version 18.2.0.37) was employed to design the molecules, simulate the cleavage of bonds, and obtain the monoisotopic masses of the molecular/fragment ions.

The mass resolution in the spectra (*m*/Δ*m*) was ∼300–400, which is not optimal. Nonetheless, it is known that mass resolution tends to be low in simple linear TOF instruments.^38^ The full width at half-maximum (fwhm) of the peaks is presented in the Supporting Information. Calibrated spectra were processed using open-source mMass software, and their intensities were normalized relative to the base peaks.

## Results and Discussion

### β-Naphthol Colorants

In the following two subsections, we present and discuss the MeV SIMS results of six samples consisting of β-naphthol colorants. The first subsection focuses on the identified pigments (**PR1** and **PR3**), while the second concentrates on the identified lakes (**PR49** and **PR53:1**). A detailed interpretation of the corresponding spectra is presented in the Supporting Information.

### β-Naphthol Pigments

**Figure 1:**
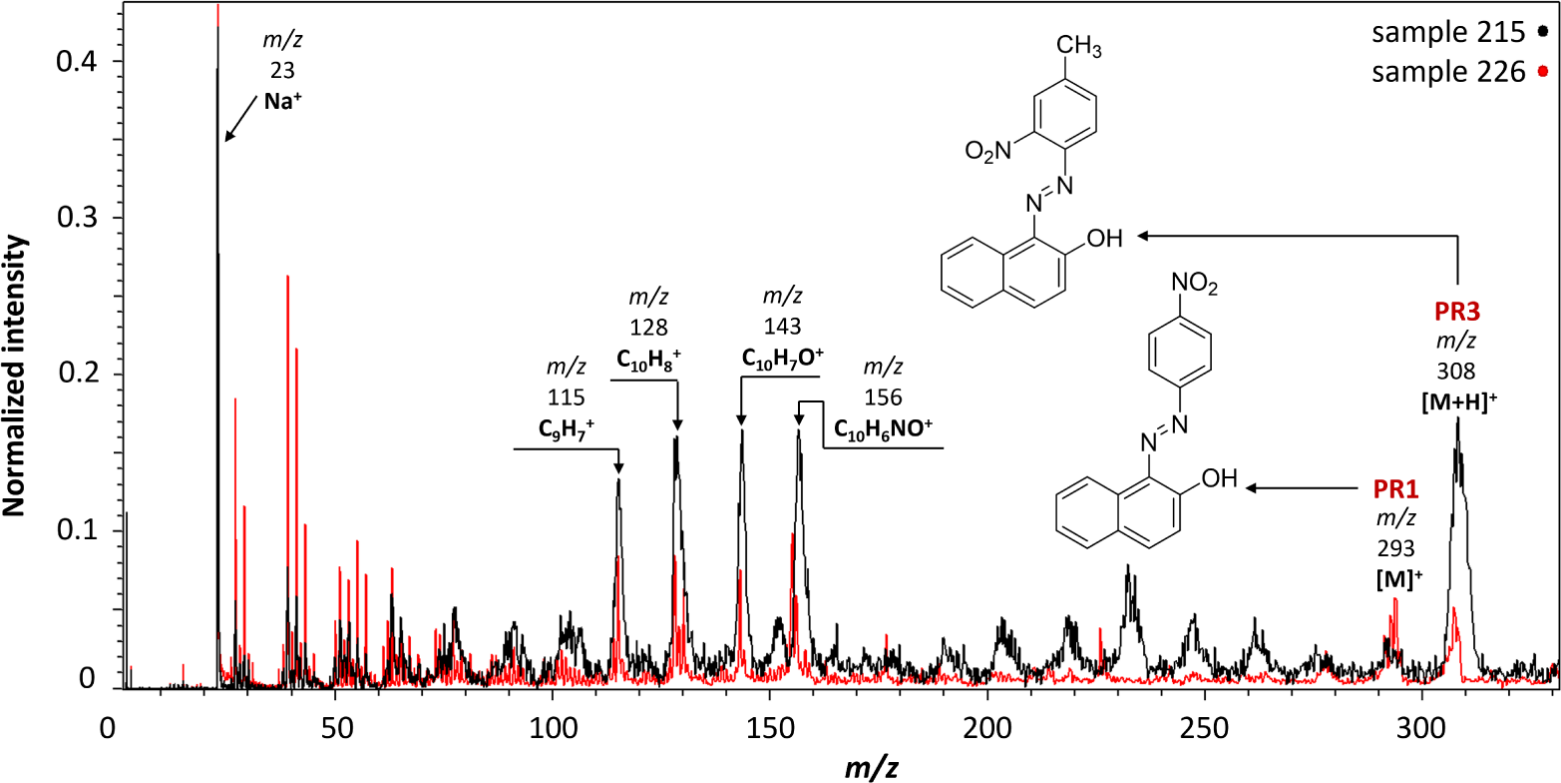
MeV SIMS spectra (5 MeV Si^4+^ positive-ion mode) of sample 215 (Monolite Fast Scarlet, I.C.I.) containing **PR3**, and sample 226 (Echtrot 1, G. Siegle & Co.) containing **PR3** and **PR1**.

Two red β-naphthol pigments were identified in the positive-ion mode, as shown in Figure 1. **PR3** was detected in four samples (215, 226, 231, and 250), all from different suppliers, with [M]^+^ at *m*/*z* 307 and [M+H]^+^ at *m*/*z* 308. Alongside **PR3**, **PR1** was identified in sample 226 due to its molecular ion [M]^+^ at *m*/*z* 293, instead of the expected PR4 suggested by the aforementioned multianalytical approach. The molecular ion [M]^+^ of PR4, having a Cl substituent on the benzene ring, if present, would have been observed at *m*/*z* 327/329. Other species characteristic of β-naphthols were also detected in the spectra of all four samples mentioned above. The characteristic 2-naphthol fragment (C_10_H_7_O^+^) was detected at *m*/*z* 143, accompanied by two other fragment ions, naphthalene (C_10_H_8_^+^) at *m*/*z* 128 and indene (C_9_H_7_^+^) at *m*/*z* 115. Although *m*/*z* 115 corresponds to In^+^, which was used as the sample support, the possibility of its detection was ruled out in this case due to the following reasons: (1) the intensity ratio to the molecular ion peak was found to be relatively constant in all samples; (2) the spatial distribution across the scanned area matched the distribution of the molecular ion species; and (3) it was not observed in the mass spectra of other colorants. Moreover, a peak at *m*/*z* 156 attributed to C_10_H_6_NO^+^ was detected in all samples.^29^

In negative-ion mode, the molecular ion [M]^−^ at *m*/*z* 307 and the deprotonated molecule [M–H]^−^ at *m*/*z* 306 of **PR3** were detected in samples 215, 231, and 250. Furthermore, characteristic functional groups were identified such as NO_2_^–^ at *m*/*z* 46 (samples 215, 231, and 250) and an additional fragment ion (C_6_H_3_NO_2_^–^) at *m*/*z* 121 (sample 215). However, no ions were identified in the negative-ion mode for sample 226, most probably due to sample charging as our setup is not equipped with a flood gun for charge compensation.

### β-Naphthol Lakes

**Figure 2:**
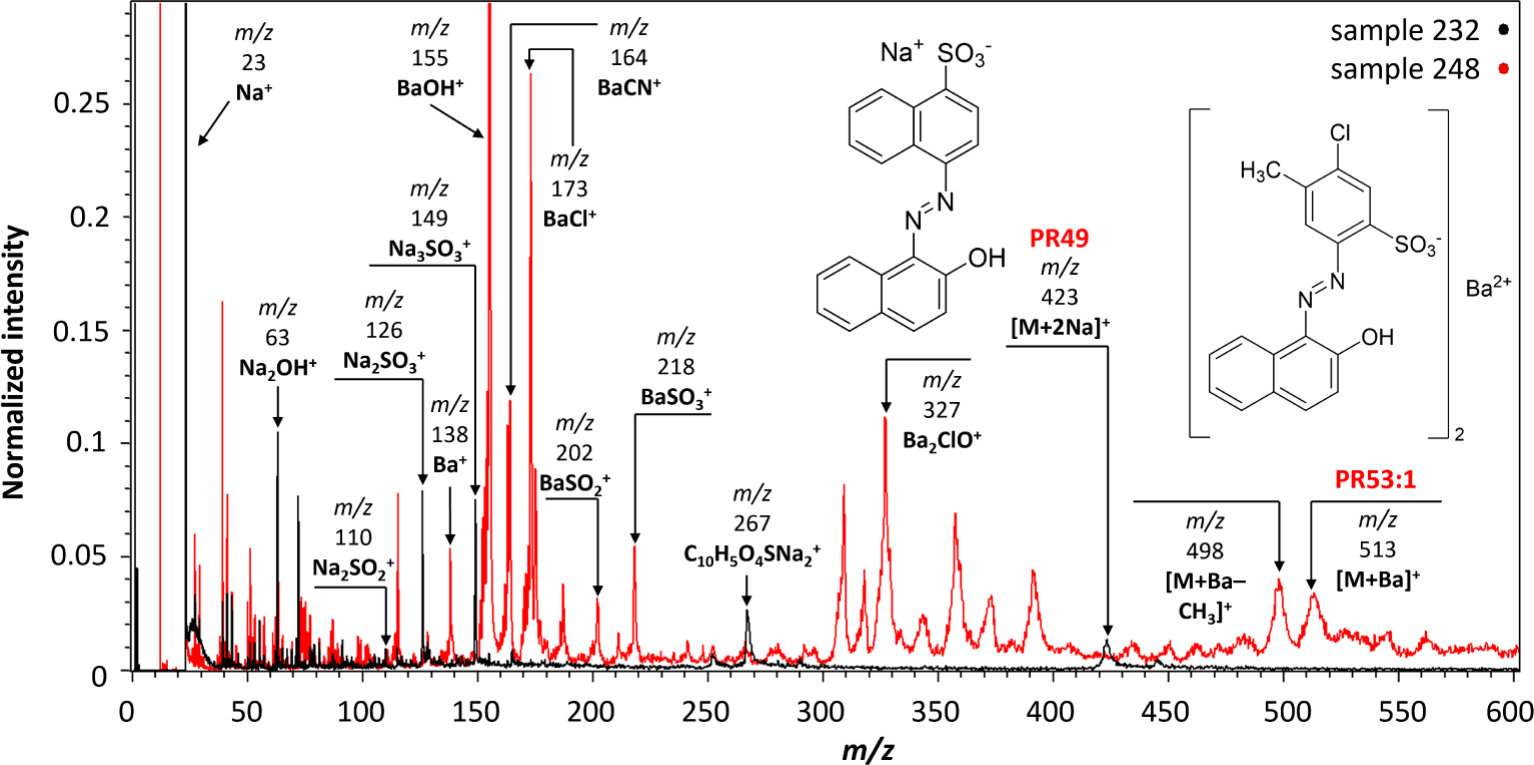
MeV SIMS spectra (5 MeV Si^4+^ positive-ion mode) of sample 232 (Lithol Red R 4593, unknown manufacturer/reseller) containing **PR49** and sample 248 (Spektralrot gelbl. Extr., Kast + Ehinger) containing **PR53:1**.

Two β-naphthol lake adduct ions were observed in positive-ion mode, specifically **PR49** (sample 232) and **PR53:1** (sample 248), as depicted in Figure 2. Notably, the mass spectra exhibited an unexpected dominance of ions, which represented inorganic species. The presence of sodium and barium within the organic structures of **PR49** and **PR53:1**, and not separately as inorganic additives (e.g., BaSO_4_), might be responsible for their detection in the spectra. Particularly, these inorganic species are bonded to the parent organic molecules and are not separate entities.

The sodium adduct [M+2Na]^+^ of **PR49** (sample 232) was observed at *m*/*z* 423, along with two fragment ions that may originate from the parent ion: *m*/*z* 252, possibly C_10_H_6_O_3_SNa_2_^+^ and *m*/*z* 267 assigned to C_10_H_5_O_4_SNa_2_^+^. The lake nature of the colorant was confirmed by the intense sodium ion peaks (Na^+^) at *m*/*z* 23 and Na_2_OH^+^ at *m*/*z* 63. Furthermore, fragment ions of the inorganic species containing sodium were also identified, namely, Na_2_SO_3_^+^ (*m*/*z* 126) and Na_3_SO_3_^+^ (*m*/*z* 149). The presence of **PR53:1** in sample 248 was confirmed by the detection of the barium adduct [M+Ba]^+^ at *m*/*z* 513, along with the fragment ion generated by the loss of a methyl group at *m*/*z* 498 [M+Ba–CH_3_]^+^. Despite its relatively low intensity, the barium ion peak (Ba^+^) was detected at *m*/*z* 138 as well as other barium-containing species. A highly intense peak was detected at *m*/*z* 155, attributed to BaOH^+^, with other lower intensity peaks at *m*/*z* 164 (BaCN^+^), 173 (BaCl^+^), 202 (BaSO_2_^+^), 218 (BaSO_3_^+^), and 327 (Ba_2_ClO^+^).

**Figure 3:**
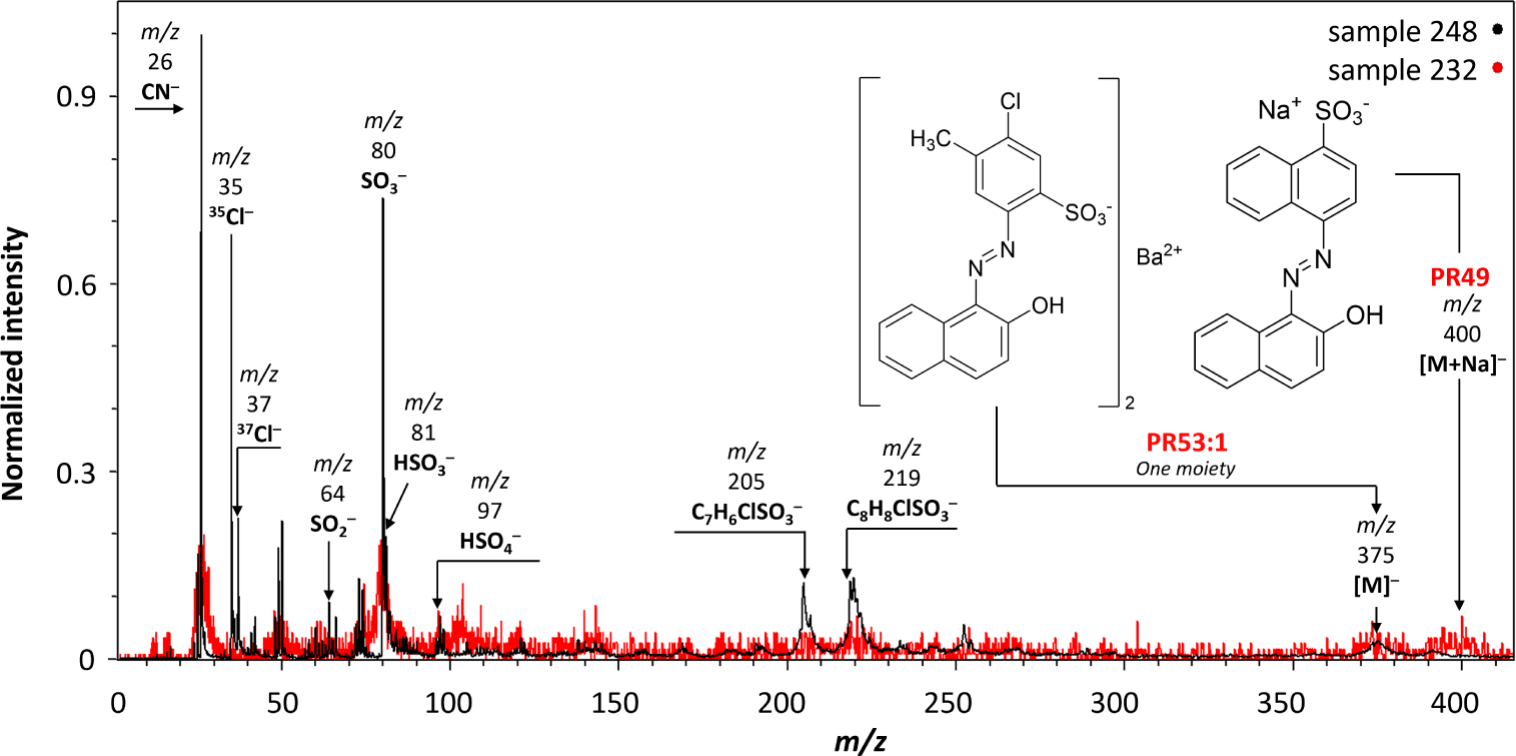
MeV SIMS spectra (5 MeV Si^4+^ negative-ion mode) of sample 232 (Lithol Red R 4593, unknown manufacturer/reseller) containing **PR49** and sample 248 (Spektralrot gelbl. Extr., Kast + Ehinger) containing **PR53:1**.

In the negative-ion mode, the molecular ion [M]^−^ of PR53:1 (sample 248) at *m*/*z* 375 was detected, representing one organic moiety, as illustrated in Figure 3. Additionally, at *m*/*z* 400, the sodium adduct [M+Na]^−^ of **PR49** was observed in sample 232 but with very low intensity. Significantly longer acquisition times would be required for better statistics, but this was not possible during our experiments due to high sample load for the designated beamtime. However, its identification is supported by its previous detection in the negative-ion mode by laser desorption ionization mass spectrometry (LDI-MS).^42^ Other characteristic negative ions with high intensities were also identified, including SO3^–^ (*m*/*z* 80) in samples 232 and 248 and the two isotopes of chlorine (^35^Cl^–^ and ^37^Cl^–^) in sample 248.

### Triarylcarbonium Colorants

In the following four subsections, we present and discuss the MeV SIMS results of analyzed triarylcarbonium colorants (25 samples in total). In the first three subsections, triarylcarbonium toners determined in the positive-ion mode are grouped by color: red (**PR81**), violet and blue (**PV3**, **PV39**, **PB1**, **PB2**, **PB3**, and **PB10**), and green (**PG1** and **PG2**). The last subsection highlights the identification of heteropolyacids in these samples, detected in negative-ion mode (PTMA, PMA). Detailed interpretation of the corresponding spectra is presented in the Supporting Information.

### Red Triarylcarbonium Toner

**Figure 4:**
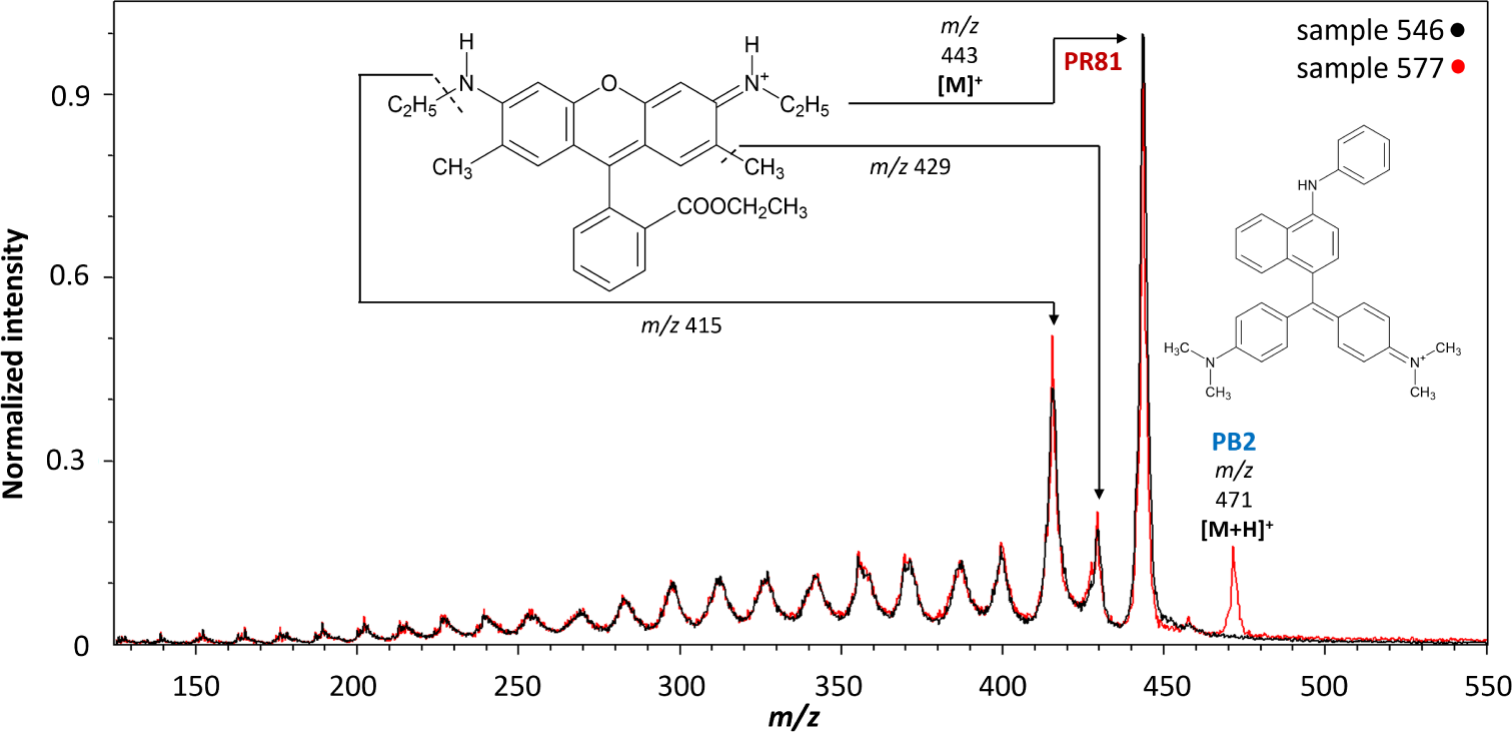
MeV SIMS spectra (5 MeV Si^4+^ positive-ion mode) of sample 546 (Brillfast Red 6114, J.W.and T.A. Smith) containing **PR81** and sample 577 (Fastel Pink 2B Supra Powder, I.C.I.) containing **PR81 and PB2**.

The molecular ion [M]^+^ at *m*/*z* 443 of **PR81** was identified in the spectra of samples 489, 546, 577, and 867. The mass spectra of samples 546 and 577 are shown in Figure 4. The fragmentation pattern of **PR81** resembled that of its corresponding dye, Rhodamine 6G.^43^ Its main three fragment ions were found at *m*/*z* 429, representing the loss of a methyl group and replacement with a hydrogen atom (−14 Da); *m*/*z* 415, the most abundant fragment ion, indicating the loss of an ethyl group and its replacement with a hydrogen atom (−28 Da); and *m*/*z* 399, demonstrating the loss of a 2-aminoethyl group (−44 Da) without replacement. Furthermore, the low-intensity peak at *m*/*z* 399 aided in differentiating between **PR81** and PV1. Although both compounds have the same molecular mass, PV1, which is the precipitation product of the Rhodamine B dye, is distinguished by its intense fragment ion at *m*/*z* 399.^43^ Additionally, PV1 lacks an intense peak at *m*/*z* 415, which is the main fragment ion of **PR81**. Despite this, the presence of this violet toner in the mixture could not be completely excluded. Many other fragment ions of **PR81** were found, ranging from *m*/*z* 139 to 387. These were probably generated by demethylation/deethylation processes. Two red powders, samples 489 and 577, were also included **PB2**, whose protonated molecule was identified at *m*/*z* 471.

### Violet and Blue Triarylcarbonium Toners

**Figure 5:**
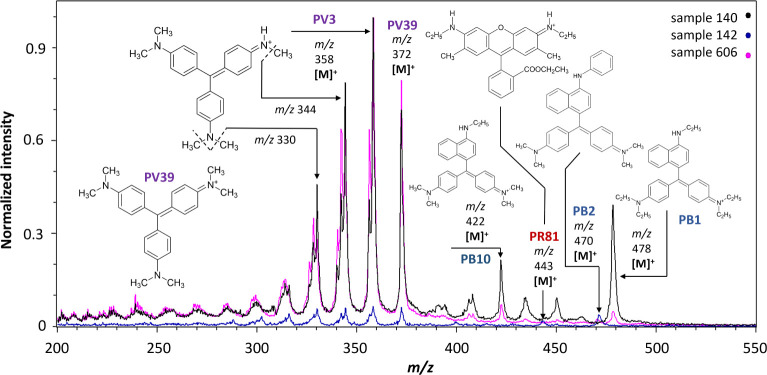
MeV SIMS spectra (5 MeV Si^4+^ positive-ion mode) of sample 140 (Violett 62 492 N 140, G. Siegleand Co.) with **PV3**, **PV39**, **PB1**, and **PB10**; sample 142 (Rotviolett D 447, G. Siegleand Co.) with **PV3**, **PV39**, **PR81**, and **PB2**; and sample 606 (Irgalite Violet TCR, Geigy) with **PV3**, **PV39**, **PB1**, **PB2**, and **PB10**.

All examined violet samples (140, 141, 142, 144, 503, 513, 573, and 606) contained **PV3** and/or **PV39**. **PV3** and **PV39** share similar molecular masses and structures.^11^**PV3** is a precipitate of Methyl Violet and is a pentamethyl triarylcarbonium toner frequently used in pen inks.^40^ In contrast, **PV39** is derived from Crystal Violet, a widely used dye in ballpoint ink production,^41^ and is a hexamethyl triarylcarbonium toner. Both **PV3** and **PV39** are characterized by the same molecular and fragment ions as those of their corresponding dye(s). All eight samples presented highly intense peaks at *m*/*z* 358, corresponding to the [M]^+^ of **PV3**, and at *m*/*z* 372, representing the [M]^+^ of **PV39**. Therefore, it is presumed that both toners have been used. This result contradicts the data from I.C.I., the producer of the Fastel line, which claimed that their violet toners, such as Fastel Violet R Supra (sample 573), consisted solely of **PV3**.^44^ Moreover, **PV3** and **PV39** have identical degradation (natural aging) and fragmentation pathways by demethylation, so the main species detected in the mass spectra correspond to the molecular ions of **PV3**/**PV39** colorants and their degradation products.^45^ Due to the low mass resolution of our linear TOF spectrometer, the characteristic fragments of **PV3**/**PV39** and their demethylated derivatives could not be fully resolved. Multiple peaks detected at *m*/*z* 356–358, 340–344, 326–330, and 312–316 (centroids at 358, 344, and 330 in Figure 5) actually correspond to the characteristic fragments of **PV3** ([M–CH_3_]^+^ at *m*/*z* 343, [M–CH_4_]^+^ at *m*/*z* 342, [M–2CH_4_]^+^ at *m*/*z* 326, etc.) and **PV39** ([M–CH_3_]^+^ at *m*/*z* 357, [M–CH_4_]^+^ at *m*/*z* 356, [M–2CH_4_]^+^ at *m*/*z* 340, etc.), as well as to their tetramethyl ([M]^+^ at *m*/*z* 344, [M–CH_3_]^+^ at *m*/*z* 329, and [M–CH_4_]^+^ at *m*/*z* 328), trimethyl ([M]^+^ at *m*/*z* 330), and dimethyl ([M]^+^ at *m*/*z* 316) analogs.^40^

Blue and/or red toners were also identified in four violet samples, 140, 142, 144, and 606. Three blue triarylcarbonium toners were detected (Figure 5): **PB1** (samples 140 and 606), **PB2** (samples 142, 144, and 606), and **PB10** (samples 140 and 606). **PB1** is derived from Basic Blue 7, a dye often found alongside Crystal Violet in contemporary pen inks.^41^ The molecular ion [M]^+^ of **PB1** (*m*/*z* 478) was observed in the positive-ion mode spectra, along with its main fragment ion (*m*/*z* 450), corresponding to the loss of an ethyl group and its replacement with a hydrogen atom (−28 Da).^46^**PB2** is derived from Basic Blue 26, another dye frequently mixed with Crystal Violet, and its protonated molecule [M+H]^+^ at *m*/*z* 471 was identified in the spectra. Moreover, the molecular ion [M]^+^ of **PB10**, whose corresponding dye is Basic Blue 11, was observed at *m*/*z* 422. However, the peak at *m*/*z* 422 might also be attributed to the fragment ion of **PB1**, specific to the loss of two ethyl groups and their replacement with one hydrogen atoms each (−56 Da).^46^ Despite this, the peak was assigned to **PB10** due to its high intensity relative to the molecular ion of **PB1**.^28^ In sample 142 (red violet; Rotviolett D447), the low-intensity molecular ion [M]^+^ of **PR81** was detected at *m*/*z* 443 together with **PV3**, **PV39**, and **PB2**.

**Figure 6:**
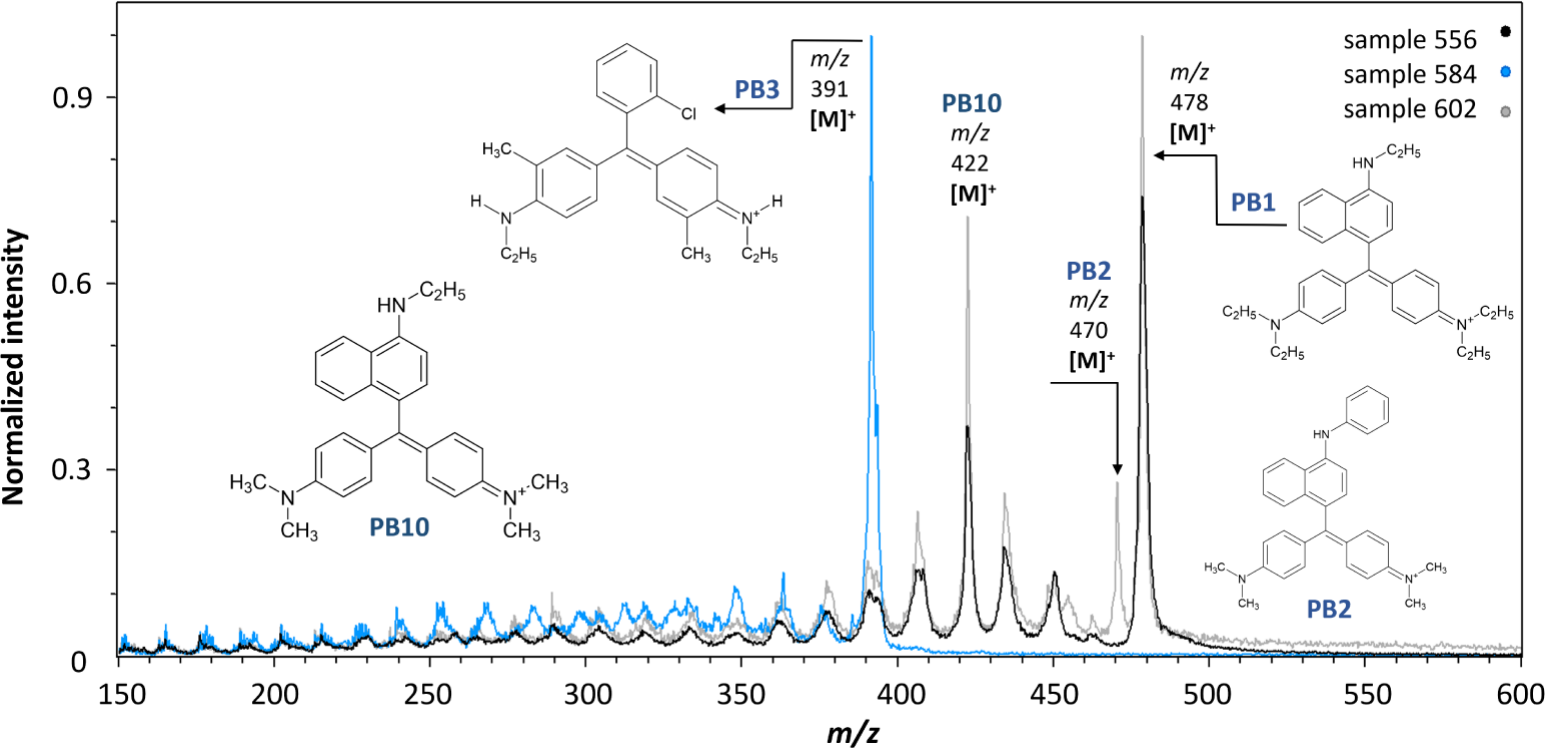
MeV SIMS spectra (5 MeV Si^4+^ positive-ion mode) of sample 556 (Fastel Blue B Supra Powder, I.C.I.) with **PB1** and **PB10**, sample 584 (Irgalite azur blue TCR, Geigy) with **PB3**, and sample 602 (Irgalite Blue TCS, Geigy) with **PB1**, **PB2**, and **PB10**.

Blue toners were determined in samples 504, 556, 584, 602, 623, and 624. The main identified toners are shown in the spectra of three samples (556, 584, and 602) presented in Figure 6. The same blue toners identified in the violet powders (Figure 5) were also found here, and they were mostly present in colorant mixtures. Four of the samples (556, 602, 623, and 624) contained two to four colorants. **PB1** was present in all four samples, as indicated by the detection of its molecular and main fragment ions (Figure 6). Along with **PB1**, the spectra of samples 602, 623, and 624 included the molecular and main fragment ions of **PB2**. Furthermore, **PB10** was found in samples 556, 602, and 624. Sample 623 contained **PB3**, together with **PB1** and **PB2**. **PB3** is derived from the triarylmethane dye Basic Blue 5, and its presence was shown by its molecular ion [M]^+^ (*m*/*z* 391), as well as three fragment ions (*m*/*z* 375, 363, and 348). Although no references were found in the literature outlining the fragmentation pattern of **PB3**, we propose that the first fragment ion (*m*/*z* 375) represents the loss of one methyl group along with one hydrogen atom (−16 Da); the second one (*m*/*z* 363) corresponds to the loss of one ethyl group and its replacement with a hydrogen atom (−28 Da); and the third one (*m*/*z* 348) represents the loss of a 2-aminoethyl group (−43 Da). Only two samples (504 and 584) contained a single blue toner. The former contained **PB1** and the latter contained **PB3**. Numerous fragment ions were observed in all blue toners (see the Supporting Information). Although attempts have been made to assign each fragment ion to a specific toner, these efforts were unsuccessful due to the presence of colorant mixtures and similar toner structures. Nonetheless, colorants could be identified through the molecular ion and two or three larger fragment ions.

### Green Triarylcarbonium Toners

Green triarylcarbonium toners were detected in samples 411, 450, 464, 537, 594, 595, and 627. All of these samples contained **PG1**, whose corresponding dye is Diamond Green G (Basic Green 1). Its molecular ion (*m*/*z* 386) and fragment ions were identified. The fragmentation pattern of **PG1** appeared to be characterized by deethylation/demethylation. The first fragment ion was observed at *m*/*z* 358 (−28 Da), indicating the loss of one ethyl group and its replacement with a hydrogen atom. The second ion appeared at *m*/*z* 342 (−44 Da), likely resulting from the loss of one ethyl and one methyl group. The third ion at *m*/*z* 328 (−58 Da) suggests the loss of two ethyl groups.

**Figure 7:**
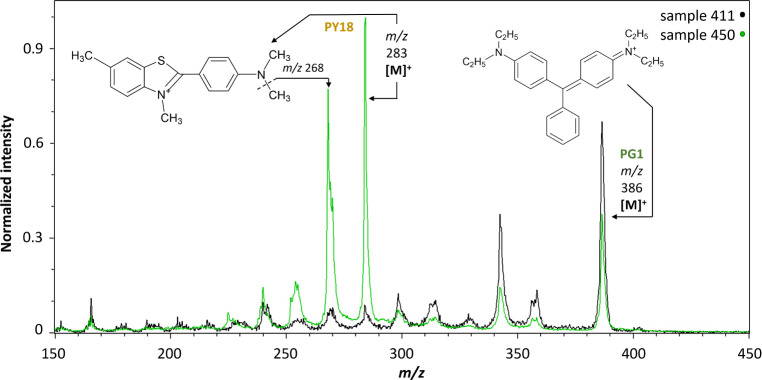
MeV SIMS spectra (5 MeV Si^4+^ positive-ion mode) of sample 411 (Fanalgrün, I.G. Farben) containing **PG1** and sample 450 (Spektraltiefgrün gelbl. 2320, Kast + Ehinger) containing **PG1** and **PY18**, which marks the presence of **PG2**.

MeV SIMS spectra indicated that four of the green powder samples, 450, 464, 537, and 594, also contained a yellow pigment. The molecular ion detected at *m*/*z* 283 suggests the presence of **PY18** in samples 450, 537, and 594, a pigment derived from thioflavine and obtained by the precipitation with PTMA.^18^ This molecular ion peak overlaps with the fragment ion of PG1 at *m*/*z* 284. However, its relative intensity was substantially lower in the case of PG1, as shown in Figure 7, which indicated that an additional colorant was present in these samples. It seems that another yellow synthetic organic pigment that was unidentifiable so far was present in sample 464, probably a colorant from diarylide class (peaks at *m*/*z* 397, 409, and 451). However, identifying yellow SOPs besides PG1^11^ implied that **PG2** was present in all four samples (450, 464, 537, and 594).

### Heteropolyacids in Triarylcarbonium Toners

**Figure 8:**
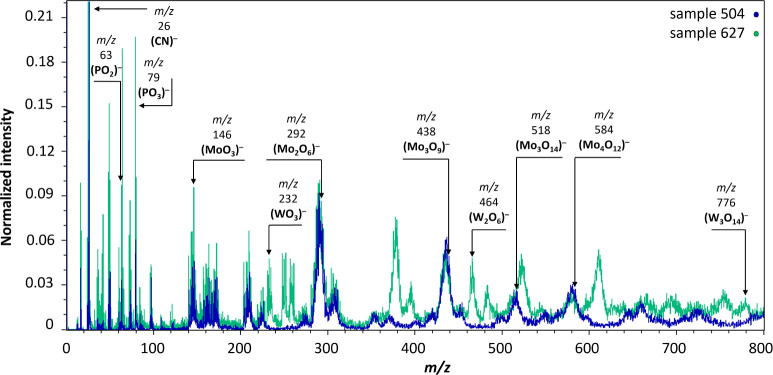
MeV SIMS spectra (5 MeV Si^4+^ negative-ion mode) showing the heteropolyacids PMA and PTMA present in samples 504 (Climatone Blue Toner, J.S.& W.R. Eakins) with PMA and 627 (Brillfast Deep Green, J.W. & T.A. Smith) with PMA and PTA, signaling the presence of PTMA.

The species corresponding to the heteropolyacids in the triarylcarbonium toners were detected only in the negative-ion mode. Negative secondary polyatomic ions such as PO_2_^–^ (*m*/*z* 63), PO_3_^–^ (*m*/*z* 79), H_2_PO_4_^–^ (*m*/*z* 97), MoO_3_^–^ (*m*/*z* 146), WO_3_^–^ (*m*/*z* 232), Mo_2_O_6_^–^ (*m*/*z* 292), and W_2_O_6_^–^ (*m*/*z* 464) were identified. However, their intensities were relatively low. Similar to the findings of Minenkova et al.,^47^ both PTMA and PMA exhibited fragment and cluster ions mainly in the form of (WO_3_)_*n*_^–^ and (MoO_3_)_*n*_^–^, respectively. By identifying PO_3_^–^, MoO_3_^–^, and WO_3_^–^ species in the spectra, PTMA’s presence was confirmed as a precipitating agent.^11^ PTMA was detected in most triarylcarbonium colorants, specifically in pigment lines such as Fanal (I.G. Farben), Siegle D (Siegle),^14^ and Fastel (I.C.I.).^44^ PMA was identified as a precipitating agent in three toners (samples 503, 504, and 513), supported by the presence of molybdenum species and absence of tungsten species. The molecular weights of PMA species identified by Minenkova et al.^47^ align with the *m*/*z* values of the species identified in the SOCs from the INTK collection. These three PMA colorants were all manufactured by J.S. & W.R. Eakins, suggesting a preference for PMA by this supplier. However, PMA colors are considered inferior to PTMA-based ones due to poorer lightfastness.^6^ The ability to distinguish between PTMA and PMA by MeV SIMS is demonstrated in Figure 8.

Furthermore, recognizing heteropolyacids is necessary for distinguishing between toners with the same molecular structure but prepared with different precipitating agents. For instance, the presence of **PV3** instead of PV27 was confirmed only in the negative-ion mode as these two toners share the same molecular formula (C_24_H_28_N_3_) but differ in precipitating agents. **PV3** contains PTMA or PMA, while PV27 contains copper ferrocyanide (CF). Since only PTMA or PMA were identified, the presence of PV27 was excluded. Nonetheless, in cases of colorant mixtures, it is currently impossible to determine whether a toner was mixed with a dye (no heteropolyacids in the spectra) or with another toner precipitated using, e.g., single agents (e.g., PMA and PTA) and not a combined variant (PTMA).

### Overview

A concise summary of the MeV SIMS findings in contrast to those from the multianalytical study (XRF, FTIR, and micro-Raman; unpublished data) on the same samples is presented in Table 2.

**Table 2:** Colorants Identified with XRF, FTIR,^37^ and Micro-Raman in Previous Work and with MeV SIMS in this Work

Inv. no. = sample	Chemical (sub)class	Colorants by XRF, FTIR, and micro-Raman (in **bold**) and precipitating agents (if present)	Colorants by MeV SIMS (in **bold**) and precipitating agents (if present)
140	triarylcarbonium toner	**PV3** (PTMA)	**PV3**, **PV39**, **PB1**, **PB10** (PTMA)
141	triarylcarbonium toner	**PV3** (PTMA)	**PV3** and **PV39** (PTMA)
142	triarylcarbonium toner	**PV3** (PTMA)	**PV3**, **PV39**, **PB2**, and **PR81**
144	triarylcarbonium toner	**PV3** (PTMA)	**PV3**, **PV39**, and **PB2**
215	β-naphthol pigment	**PR3**	**PR3**
226	β-naphthol pigment	**PR1** or **PR4**	**PR1**, **PR3**
231	β-naphthol pigment	**PR3**	**PR3**
232	β-naphthol lake	**PR49**	**PR49**
248	β-naphthol lake	**PR53:1**	**PR53:1**
250	β-naphthol pigment	**PR3**	**PR3**
411	triarylcarbonium toner	**PG1** (PTMA)	**PG1** (PTMA)
450	triarylcarbonium toner	**PG1** or **PG2** or **PG4** (PTMA)	**PG2** (PTMA)
464	triarylcarbonium toner	**PG1**or **PG2** or **PG4** (PTMA)	**PG2** (PG1 + diarylide yellow) (PTMA)
489	triarylcarbonium toner	**PR81** (PTMA)	**PR81** and **PB2** (PTMA)
503	triarylcarbonium toner	**PV3** (PMA)	**PV3** and **PV39** (PMA)
504	triarylcarbonium toner	**PB1** (PMA)	**PB1** (PMA)
513	triarylcarbonium toner	**PV3** (PMA)	**PV3** and **PV39** (PMA)
537	triarylcarbonium toner	**PG2** (PTMA)	**PG2** (PTMA)
546	triarylcarbonium toner	**PR81** (PTMA)	**PR81** (PTMA)
556	triarylcarbonium toner	**PB1** (PTMA)	**PB1** and **PB10** (PTMA)
573	triarylcarbonium toner	**PV3** (PTMA)	**PV3** and **PV39** (PTMA)
577	triarylcarbonium toner	**PR81** (PTMA)	**PR81** and **PB2** (PTMA)
584	triarylcarbonium toner	**PB3** (PTMA)	**PB3** (PTMA)
594	triarylcarbonium toner	**PG1** or **PG2** or **PG4** (PTMA)	**PG2** (PTMA)
595	triarylcarbonium toner	**PG1** (PTMA)	**PG1** (PTMA)
602	triarylcarbonium toner	**PB1** (PTMA)	**PB1**, **PB2**, and **PB10** (PTMA)
606	triarylcarbonium toner	**PV3** (PTMA)	**PV3**, **PV39**, **PB1**, **PB2**, and **PB10** (PTA)
623	triarylcarbonium toner	**PB3** (PTMA)	**PB3**, **PB1**, and **PB2** (PTMA)
624	triarylcarbonium toner	**PB1** (PTMA)	**PB1**, **PB2**, and **PB10** (PTMA)
627	triarylcarbonium toner	**PG1** (PTMA)	**PG1** (PTMA)
867	triarylcarbonium toner	**PR81** (PTMA)	**PR81** (PTMA)

## Conclusions

The identification of the β-naphthol and triarylcarbonium colorants present in the analyzed samples from the 19th /20th century Materials Collection of the INTK was essential for their use as references in material analysis in the field of cultural heritage. SIMS with primary ions in the MeV range enables simultaneous identification of synthetic organic colorants (SOCs) in mixtures without chemical treatment or sample consumption. These along with other already mentioned advantages support its use over analytical methods such as Py-GC/MS, DTMS, MALDI-MS, and HPLC-MS. This work clearly demonstrates the benefits of SIMS with 5 MeV Si^4+^ to (1) identify multiple synthetic organic colorants (even four or five) in mixtures and (2) differentiate between pigments, lakes, toners, and dyes. Furthermore, MeV SIMS could validate and expand the information from the manufacturing companies of the colorants, such as I.C.I. (UK).

The MeV SIMS spectra of the colorants analyzed in this study will enrich the open-access INTK’s pigment database (currently under development). Furthermore, these spectra will enlarge the existing MeV SIMS database with synthetic organic pigments (SOPs) found in artists’ paints at the RBI, started during our earlier projects. Since β-naphthol and triarylcarbonium colorants tend to fade when exposed to light, the ability to identify these colorants in, for example, drawings and prints can contribute to preventive conservation strategies.

## Supporting Information Available

MeV SIMS spectra of the examined β-naphthol and triarylcarbonium colorants (PDF)
