# Supplementary material for: Beyond Artists’ Colors: A Spectral Reference Database for the Identification of β-Naphthol and Triarylcarbonium Colorants by MeV SIMS
Source: ACS Omega. 2024 Sep 11;9(38):39573–83. doi: 10.1021/acsomega.4c03634 (PMC11425608; doi:10.1021/acsomega.4c03634)
Supplement: Supplementary file 1 — ao4c03634_si_001.pdf [file ao4c03634_si_001.pdf]

SUPPORTING INFORMATION

Beyond Artists' Colors: A Spectral Reference Database for the Identification of β-Naphthol and Triarylcarbonium Colorants by MeV SIMS

Teodora Raicu[1,*], Matea Krmpotić[2], Zdravko Siketić[2], Iva Bogdanović Radović[2], Katja Sterflinger[1], Dubravka Jembrih-Simbürger[1]

[§]Institute for Natural Sciences and Technology in the Arts, Academy of Fine Arts Vienna, Augasse 2-6, Vienna, A-1090, Austria

[✩]Division of Experimental Physics, Laboratory for Ion Beam Interactions, Ruđer Bošković Institute, Bijenička cesta 54, Zagreb, HR-10000, Croatia

Table of Contents

Table S-1. The characteristic species (molecular ions, (de)protonated molecules, fragment ions) of the examined β-naphthol and triarylcarbonium toners detected with MeV SIMS (5 MeV Si^{4+} in the positive- and negative-ion modes).

Table S-2. The characteristic species grouped by the metallic salts of β-naphthol lakes and the heteropolyacids of triarylcarbonium toners detected with MeV SIMS (5 MeV Si^{4+} in the positive- and negative-ion modes).

Figure S-1. The positive- (a) and negative-ion (b) modes (5 MeV Si^{4+}) mass spectra of sample 140 (Violett 62492 N) – G. Siegle & Co.

Figure S-2. The positive- (a) and negative-ion (b) modes (5 MeV Si^{4+}) mass spectra of sample 141 (Fanalviolett R Supra) – I.G. Farben.

Figure S-3. The positive-ion mode (5 MeV Si^{4+}) mass spectrum of sample 142 (Rotviolett D 447) – G. Siegle & Co.

Figure S-4. The positive-ion mode (5 MeV Si^{4+}) mass spectrum of sample 144 (Blauviolett D 447) – G. Siegle & Co.

Figure S-5. The positive- (a) and negative-ion (b) modes (5 MeV Si^{4+}) mass spectra of sample 215 (Monolite Fast Scarlet) – I.C.I.

Figure S-6. The positive-ion mode (5 MeV Si^{4+}) mass spectrum of sample 226 (Echtrot 1) – G. Siegle & Co.

Figure S-7. The positive- (a) and negative-ion (b) modes (5 MeV Si^{4+}) mass spectrum of sample 231 (Helioechtrot 1) – Bayer.

Figure S-8. The positive- (a) and negative-ion (b) modes (5 MeV Si^{4+}) mass spectra of sample 232 (Lithol Red R 4593) – Unknown (possibly I.G. Farben).

Figure S-9. The positive- (a) and negative-ion (b) modes (5 MeV Si^{4+}) mass spectra of sample 248 (Spektralrot gelbl. Extr.) – Kast + Ehinger.

Figure S-10. The positive- (a) and negative-ion (b) modes (5 MeV Si^{4+}) mass spectra of sample 250 (Litholechtscharlach RN) – I.G. Farben.

Figure S-11. The positive- (a) and negative-ion (b) modes (5 MeV Si^{4+}) mass spectra of sample 411 (Fanalgrün) – I.G. Farben.

Figure S-12. The positive- (a) and negative-ion (b) modes (5 MeV Si^{4+}) mass spectra of sample 450 (Spektraltiefgrün gelbl. 2320) – Kast + Ehinger.

Figure S-13. The positive-ion mode (5 MeV Si^{4+}) mass spectrum of sample 464 (Sieglegrün D451) – G. Siegle & Co.

Figure S-14. The positive- (a) and negative-ion (b) modes (5 MeV Si^{4+}) mass spectra of sample 489 (Fastel Pink B Powder) – I.C.I.

Figure S-15. The positive- (a) and negative-ion (b) modes (5 MeV Si^{4+}) mass spectra of sample 503 (Dragon Purple) – J. S. & W. R. Eakins.

Figure S-16. The positive- (a) and negative-ion (b) modes (5 MeV Si^{4+}) mass spectra of sample 504 (Climatone Blue Toner) – J. S. & W. R. Eakins.

Figure S-17. The positive- (a) and negative-ion (b) modes (5 MeV Si^{4+}) mass spectra of sample 513 (Climatone Purple Toner) – J. S. & W. R. Eakins.

Figure S-18. The positive- (a) and negative-ion (b) modes (5 MeV Si^{4+}) mass spectra of sample 537 (Fastel Yellow Green GA Supra Powder) – I.C.I.

Figure S-19. The positive- (a) and negative-ion (b) modes (5 MeV Si^{4+}) mass spectra of sample 546 (Brillfast Red 6114) – J.W. & T.A. Smith Ltd. London.

Figure S-20. The positive- (a) and negative-ion (b) modes (5 MeV Si^{4+}) mass spectra of sample 556 (Fastel Blue B Supra Powder) – I.C.I.

Figure S-21. The positive- (a) and negative-ion (b) modes (5 MeV Si^{4+}) mass spectra of sample 573 (Fastel Violet R Supra Powder) – I.C.I.

Figure S-22. The positive- (a) and negative-ion (b) modes (5 MeV Si^{4+}) mass spectra of sample 577 (Fastel Pink 2B Supra Powder) – I.C.I.

Figure S-23. The positive- (a) and negative-ion (b) modes (5 MeV Si^{4+}) mass spectra of sample 584 (Irgalite azur blue TCR) – Geigy.

Figure S-24. The positive- (a) and negative-ion (b) modes (5 MeV Si^{4+}) mass spectrum of sample 594 (Vert clair Lumière) – Cappelle Frères.

Figure S-25. The positive- (a) and negative-ion (b) modes (5 MeV Si^{4+}) mass spectra of sample 595 (Vert forte Lumière) – Cappelle Frères.

Figure S-26. The positive- (a) and negative-ion (b) modes (5 MeV Si^{4+}) mass spectra of sample 602 (Irgalite Blue T C S) – Geigy.

Figure S-27. The positive- (a) and negative-ion (b) modes (5 MeV Si^{4+}) mass spectra of sample 606 (Irgalite Violet T C R) – Geigy.

Figure S-28. The positive- (a) and negative-ion (b) modes (5 MeV Si^{4+}) mass spectra of sample 623 (Brillfast Sky Blue 3862) – J.W. & T.A. Smith.

Figure S-29. The positive- (a) and negative-ion (b) modes (5 MeV Si^{4+}) mass spectra of sample 624 (Brillfast Maltese Blue 3591) – J.W. & T.A. Smith.

Figure S-30. The positive- (a) and negative-ion (b) modes (5 MeV Si^{4+}) mass spectra of sample 627 (Brillfast Deep Green) – J.W. & T.A. Smith.

Figure S-31. The positive- (a) and negative-ion (b) modes (5 MeV Si^{4+}) mass spectra of sample 867 (Fastel Pink R Supra Powder) – I.C.I.

Table S-3. Detailed results gained form previous research with XRF and Raman/FTIR alongside the MeV SIMS results.

Note: The MeV SIMS spectra outlined in green were acquired in the positive-ion mode and those in red in the negative-ion mode with a 5 MeV Si^{4+} primary ion beam (Figures S-1 – S-31).

Table S-1. The characteristic species (molecular ions, (de)protonated molecules, fragment ions) of the examined β-naphthol and triarylcarbonium toners detected with MeV SIMS (5 MeV Si^{4+} in the positive- and negative-ion modes).

samples (INTK inv. no.)	chemical (sub)class	color index name	*m/z*	FWHM	fragment ion
226	β-naphthol pigment	**PR1**	115	0.4220	$C_9H_7^+$
			128	0.4697	$C_{10}H_8^+$
			143	0.4552	$C_{10}H_7O^+$
			156	0.6702	$C_{10}H_6NO^+$
			293	0.7554	$[M]^+$
215, 226, 231, 250	β-naphthol pigment	**PR3**	115	0.4077 –1.7541	$C_9H_7^+$
			128	0.4697 –2.4348	$C_{10}H_8^+$
			143	0.4514 – 1.9016	$C_{10}H_7O^+$
			156	0.5778 – 2.1062	$C_{10}H_6NO^+$
			261	1.3541 – 2.9098	$[M–NO_2]^+$
			307, 308	1.7655 – 3.9599	$[M]^+$, $[M+H]^+$
232	β-naphthol lake	**PR49, Na salt**	252	1.7930	$C_{10}H_6O_3SNa_2^+$
			267	1.8946	$C_{10}H_5O_4SNa_2^+$
			423	2.7973	$[M+2Na]^+$
248	β-naphthol lake	**PR53:1, Ba salt**	281	0.3167	$C_{10}H_7OBa^+$
			498	5.3158	$[M+Ba–CH_3]^+$
			513	7.5240	$[M+Ba]^+$
140, 141, 142, 144, 503, 513, 573, 606	triarylcarbonium toner	**PV39**	316	1.002 –5.402	$[M+4H–4CH_3]^+$
			330	1.089 – 6.1308	$[M+3H–3CH_3]^+$
			344	0.983 – 4.7831	$[M+2H–2CH_3]^+$
			358	1.001 – 3.4014	$[M+H–CH_3]^+$
			372	0.924 – 3.0642	$[M]^+$
140, 141, 142, 144, 503, 513, 573, 606	triarylcarbonium toner	**PV3**	316	1.002 –5.402	$[M+3H–3CH_3]^+$
			330	1.089 – 6.1308	$[M+2H–2CH_3]^+$
			344	0.983 – 4.7831	$[M+H–CH_3]^+$
			358	1.001 – 3.4014	$[M]^+$
140, 504, 556, 602, 606, 623, 624	triarylcarbonium toner	**PB1**	391	4.8178 – 6.4294	$[M–C_4H_{10}N–CH_3]^+$
			406	3.8588 – 5.9312	$[M+H–C_2H_6N–C_2H_5]^+$ or $[M–C_4H_{10}N]^+$
			434	2.8808 – 4.2765	$[M–CH_3–C_2H_5]^+$ or $[M–C_2H_6N]^+$
			450	2.0118 – 4.8557	$[M+H–C_2H_5]^+$
			478	0.0800 – 2.7534	$[M]^+$

Table S-1 (continued)

**samples (INTK inv. no.)**	chemical (sub)class	color index name	*m/z*	FWHM	fragment ion
142, 144, 489, 577, 602, 606, 623, 624	triarylcarbonium toner	**PB2**	471	1.797 – 2.5514	$[M+H]^+$
			470	1.1393 – 1.6692	$[M]^+$
584, 623	triarylcarbonium toner	**PB3**	348	4.7532 – 5.0673	$[M+H–C_2H_6N]^+$
			363	1.6287 – 3.5526	$[M+H–C_2H_5]^+$
			375	3.3319 – 3.6594	$[M–H–CH_3]^+$
			391	1.1763 –1.1892	$[M]^+$
140, 556, 602, 606, 624	triarylcarbonium toner	**PB10**	408	3.8836 – 5.8590	$[M+H–CH_3]^+$
			422	1.3450 – 2.4275	$[M]^+$
142, 489, 546, 577, 867	triarylcarbonium toner	**PR81**	341	6.1630 – 7.7909	$[M+H–2C_2H_6N–CH_3]^+$
			355	5.2643 – 7.3911	$[M–2C_2H_6N]^+$
			370	5.6124 – 6.6436	$[M–C_2H_6N–C_2H_5]^+$
			387	5.6185 – 5.9918	$[M+2H–2C_2H_5]^+$
			399	1.434 – 4.5897	$[M–C_2H_6N]^+$
			415	2.1177 – 3.2635	$[M+H–C_2H_5]^+$
			429	1.5101 – 2.6265	$[M+H–CH_3]^+$
			443	1.699 – 2.3792	$[M]^+$
411, 450, 464, 537, 594, 595, 627	triarylcarbonium toner	**PG1**	242	2.6831 – 5.3198	$[M+H–2C_4H_{10}N]^+$
			255	0.4975 – 5.5238	$[M–C_4H_{10}N–2C_2H_5]^+$
			270	2.9844 – 4.5721	$[M+H–C_4H_{10}N–C_2H_5–CH_3]^+$
			298	0.8851 – 4.0147	$[M+H–2C_2H_5–2CH_3]^+$
			313	0.6506 – 6.3305	$[M–C_4H_{10}N]^+$
			328	2.4164 – 6.8468	$[M+H–2C_2H_5]^+$
			342	1.4885 – 2.6681	$[M–C_2H_5–CH_3]^+$
			358	1.4232 – 4.1988	$[M+H–C_2H_5]^+$
			386	1.2337 – 2.0226	$[M+H]^+$
450, 537, 594	triarylcarbonium toner	**PY18**	239	0.9295 – 4.1611	$[M–C_2H_6N]^+$
			253	2.2300 – 5.4448	$[M–2CH_3]^+$
			268	0.9350 – 3.0630	$[M–CH_3]^+$
			283	0.9336 – 1.1129	$[M]^+$
464	diarylide yellow	**diarylide yellow pigment (?)**	397, 409, 451	1.7400 – 3.6657	not assigned
226	naphthol AS	**naphthol AS pigment (?)**	412, 444, 460, 468	0.7211 – 2.0346	not assigned

Table S-2. The characteristic species grouped by the metallic salts of β-naphthol lakes and the heteropolyacids of triarylcarbonium toners detected with MeV SIMS (5 MeV Si^{4+} in the positive- and negative-ion modes).

samples (INTK inv. no.)	color index name	metallic salt/ heteropolyacid	*m/z*	FWHM	Fragment ion
232	PR49	Na salt	23	0.1285	Na^+
			63	0.2652	Na_2OH^+
			110	0.3726	$Na_2SO_2^+$
			126	0.4062	$Na_2SO_3^+$
			149	0.4760	$Na_3SO_3^+$
248	PR53:1	Ba salt	138	0.5549	Ba^+
			155	0.6572	$BaOH^+$
			164	0.7668	$BaCN^+$
			173	0.7336	$BaCl^+$
			218	0.8679	$BaSO_3^+$
			327	1.8755	Ba_2ClO^+
140, 141, 411, 450, 464, 489, 503, 504, 513, 537, 546, 556, 573, 577, 584, 595, 602, 606, 623, 624, 627, 867	PV3, PV39, PB1, PB2, PB3, PB10, PG1, PY18, PR81	phosphomolybdic acid (PMA)	63	0.1458 – 0.3686	PO_2^-
			79	0.2309 – 0.6818	PO_3^-
			146	0.2749 – 1.6511	MoO_3^-
			292	0.3101 – 0.8158	$Mo_2O_6^-$
			438	0.3608 – 7.9015	$Mo_3O_9^-$
			518	0.2610 – 6.0692	$Mo_3O_{14}^-$
			584	0.2129 – 2.0247	$Mo_4O_{12}^-$
140, 141, 411, 450, 464, 489, 537, 546, 556, 573, 577, 584, 595, 602, 606, 623, 624, 627, 867	PV3, PV39, PB1, PB2, PB3, PB10, PG1, PY18, PR81	phosphotungstic acid (PTA)	63	0.2349 – 0.3686	PO_2^-
			79	0.2532 – 0.6818	PO_3^-
			232	0.2254 – 2.9811	WO_3^-
			464	0.1988 – 5.8565	$W_2O_6^-$
			696	1.8164 – 7.9083	$W_3O_9^-$
			776	0.3813 – 7.8711	$W_3O_{14}^-$
			1160	0.3301 – 1.1404	$W_5O_{15}^-$
			1392	0.8405 – 1.5353	$W_6O_{18}^-$
			2881	0.8070 – 1.7628	$P(W_3O_{10})_4^{3-}$

Figure S-1. The positive- (a) and negative-ion (b) modes (5 MeV Si^{4+}) mass spectra of sample 140 (Violett 62492 N) – G. Siegle & Co.

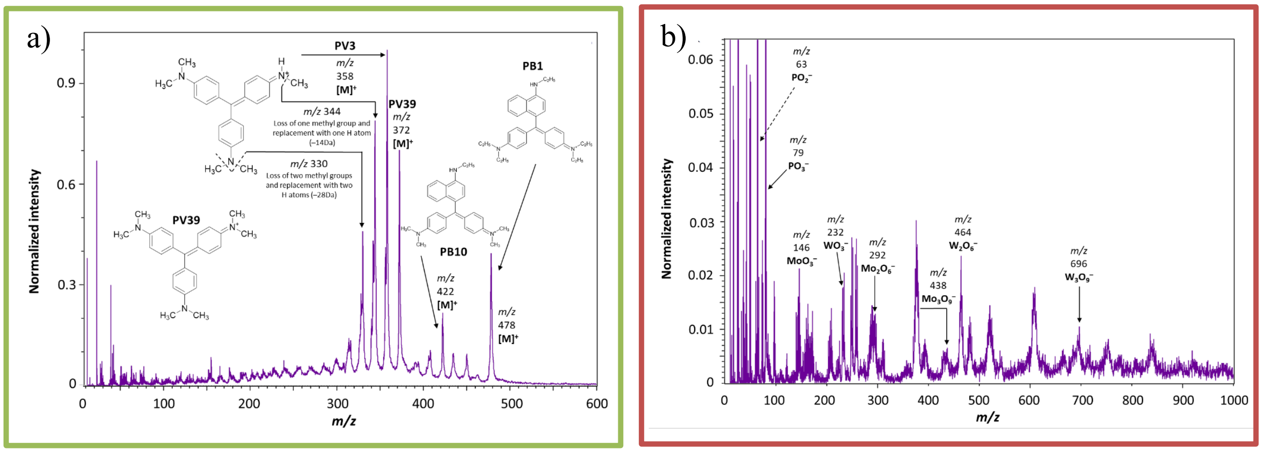

Figure S-2. The positive- (a) and negative-ion (b) modes (5 MeV Si^{4+}) mass spectra of sample 141 (Fanalviolett R Supra) – I.G. Farben.

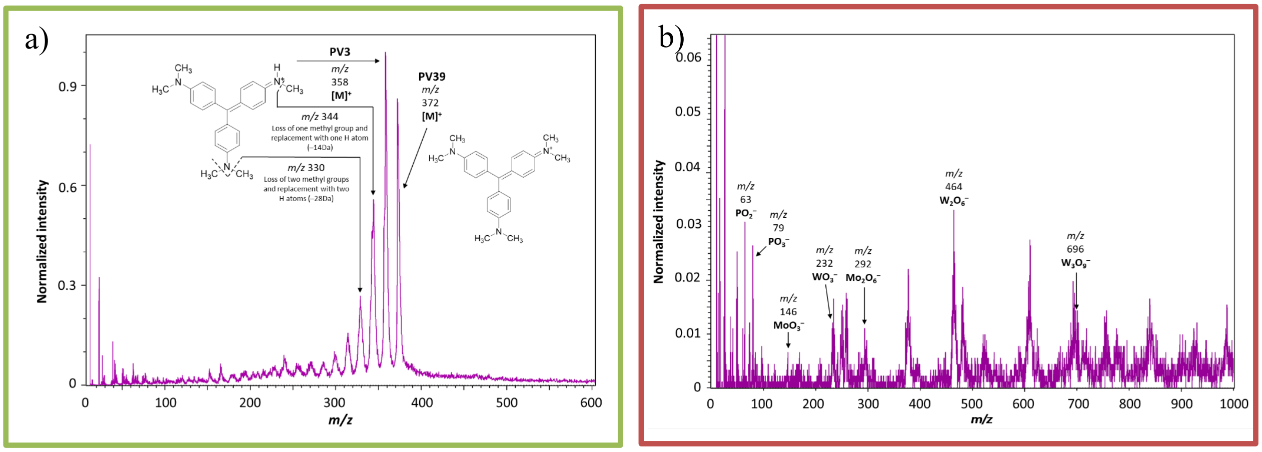

Figure S-3. The positive-ion mode (5 MeV Si^{4+}) mass spectrum of sample 142 (Rotviolett D 447) – G. Siegle & Co.

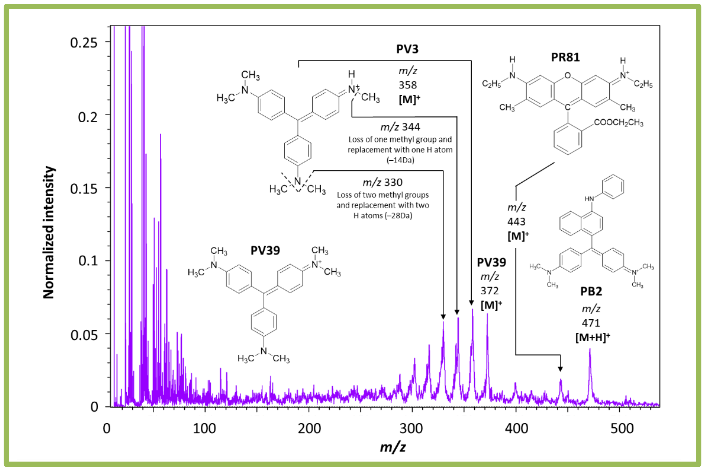

Figure S-4. The positive-ion mode (5 MeV Si^{4+}) mass spectrum of sample 144 (Blauviolett D 447) – G. Siegle & Co.

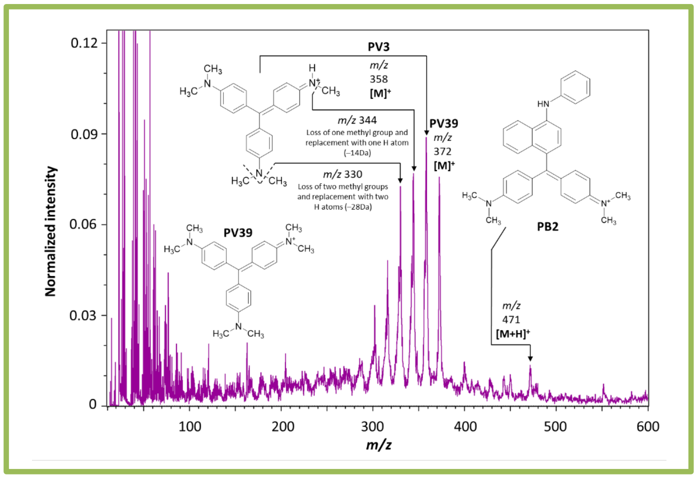

Figure S-5. The positive- (a) and negative-ion (b) modes (5 MeV Si^{4+}) mass spectra of sample 215 (Monolite Fast Scarlet) – I.C.I.

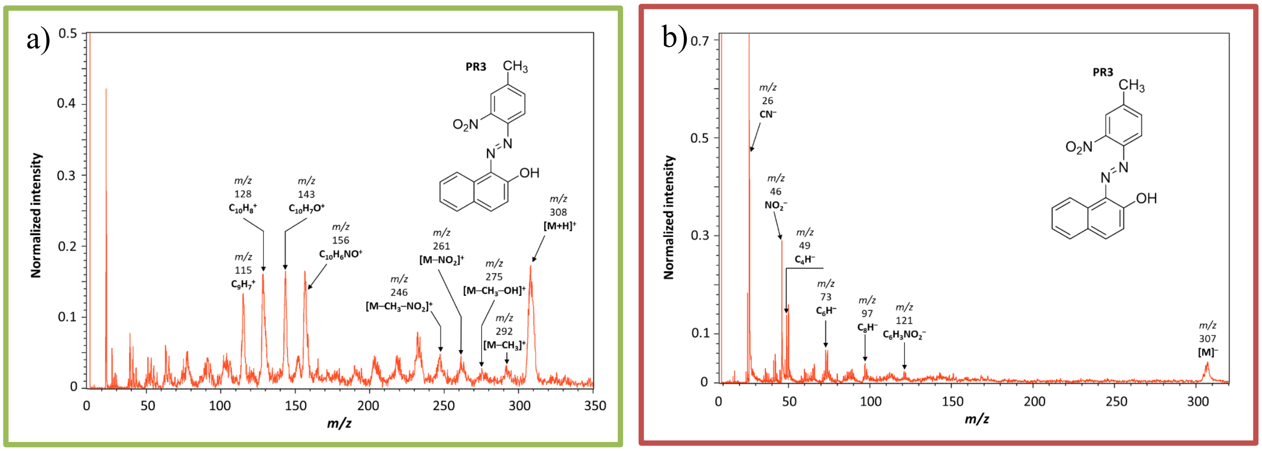

Figure S-6. The positive-ion mode (5 MeV Si^{4+}) mass spectrum of sample 226 (Echtrot 1) – G. Siegle & Co.

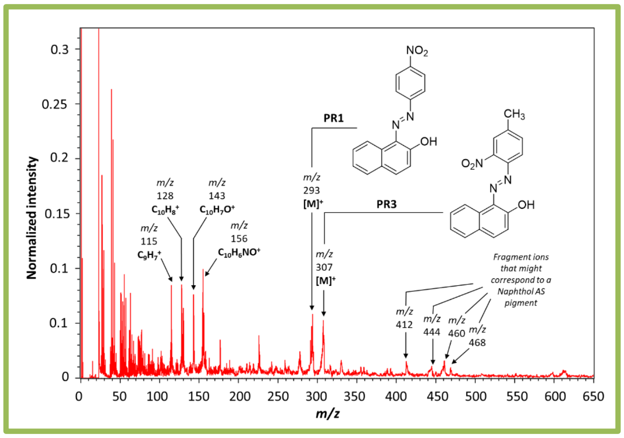

Figure S-7. The positive- (a) and negative-ion (b) modes (5 MeV Si^{4+}) mass spectrum of sample 231 (Helioechtrot RL) – Bayer.

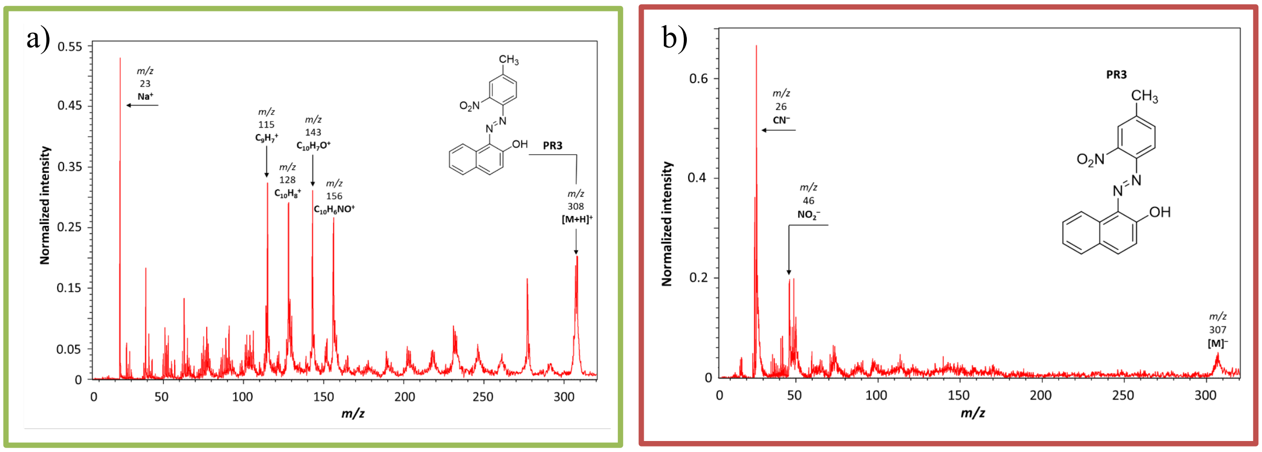

Figure S-8. The positive- (a) and negative-ion (b) modes (5 MeV Si^{4+}) mass spectra of sample 232 (Lithol Red R 4593) – Unknown.

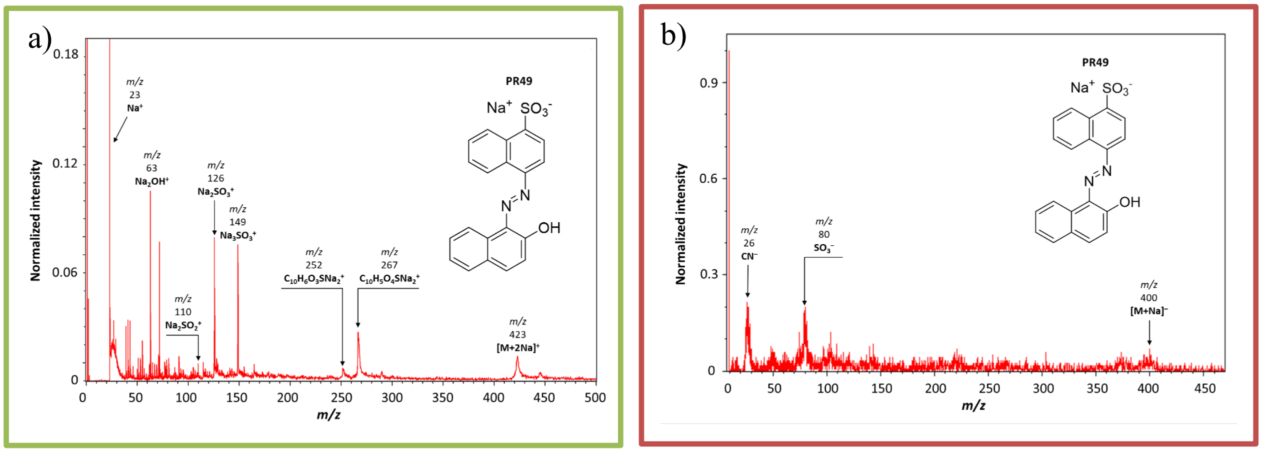

Figure S-9. The positive- (a) and negative-ion (b) modes (5 MeV Si^{4+}) mass spectra of sample 248 (Spektralrot gelbl. Extr.) – Kast + Ehinger.

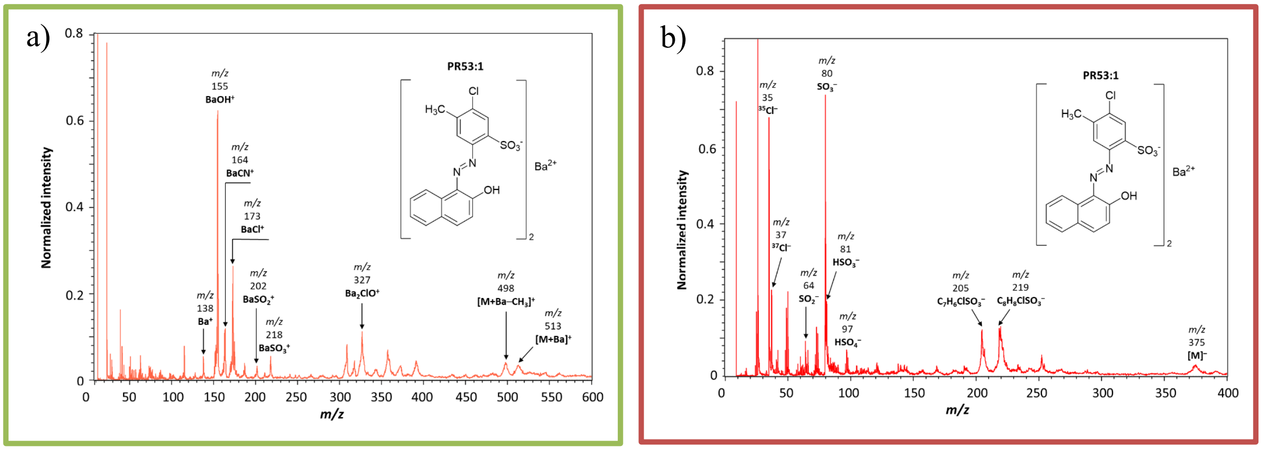

Figure S-10. The positive- (a) and negative-ion (b) modes (5 MeV Si^{4+}) mass spectra of sample 250 (Litholechtscharlach RN) – I.G. Farben.

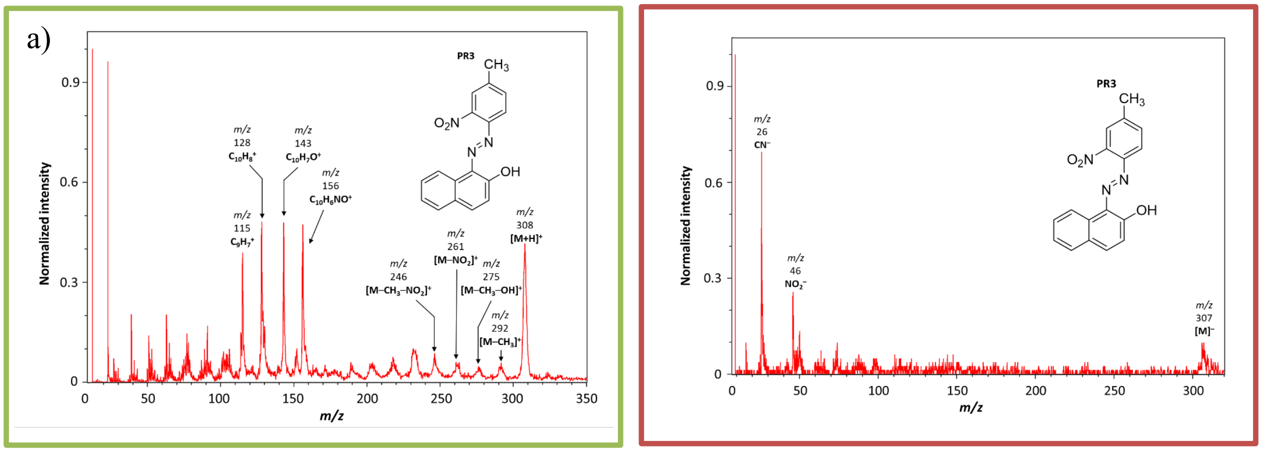

Figure S-11. The positive- (a) and negative-ion (b) modes mass spectra of sample 411 (Fanalgrün) – I.G. Farben.

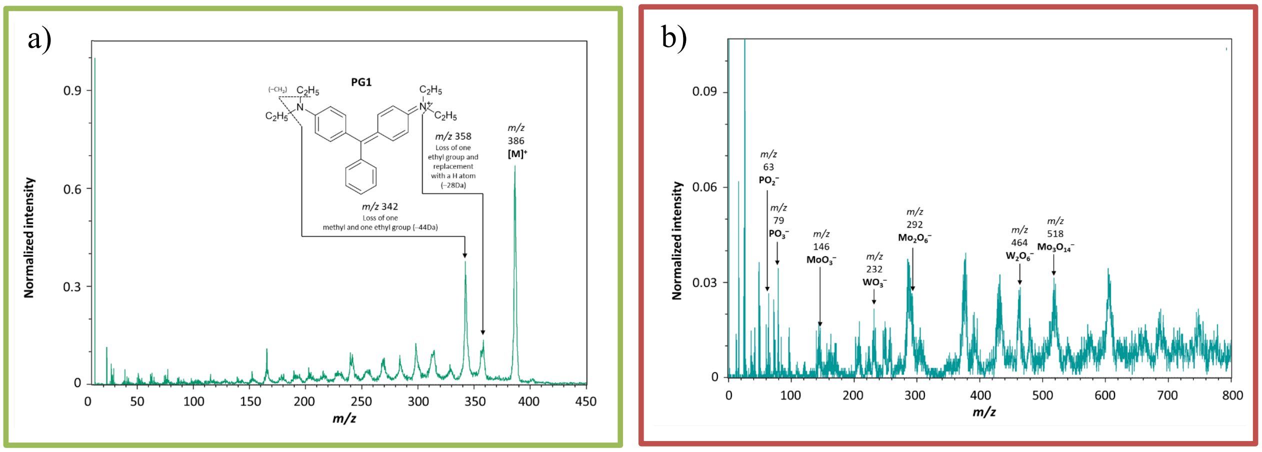

Figure S-12. The positive- (a) and negative-ion (b) modes (5 MeV Si^{4+}) mass spectra of sample 450 (Spektraltiefgrün gelbl. 2320) – Kast + Ehinger.

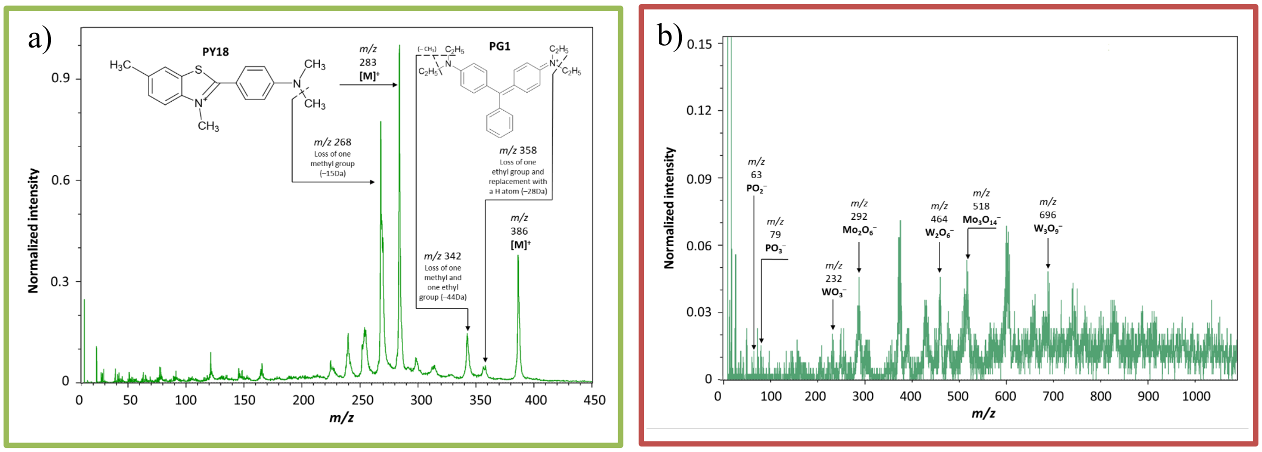

Figure S-13. The positive-ion mode (5 MeV Si^{4+}) mass spectrum of sample 464 (Sieglegrün D451) – G. Siegle & Co.

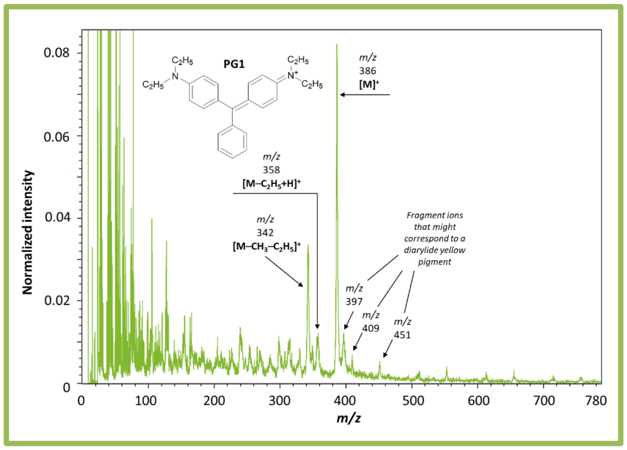

Figure S-14. The positive- (a) and negative-ion (b) modes (5 MeV Si^{4+}) mass spectra of sample 489 (Fastel Pink B Powder) – I.C.I.

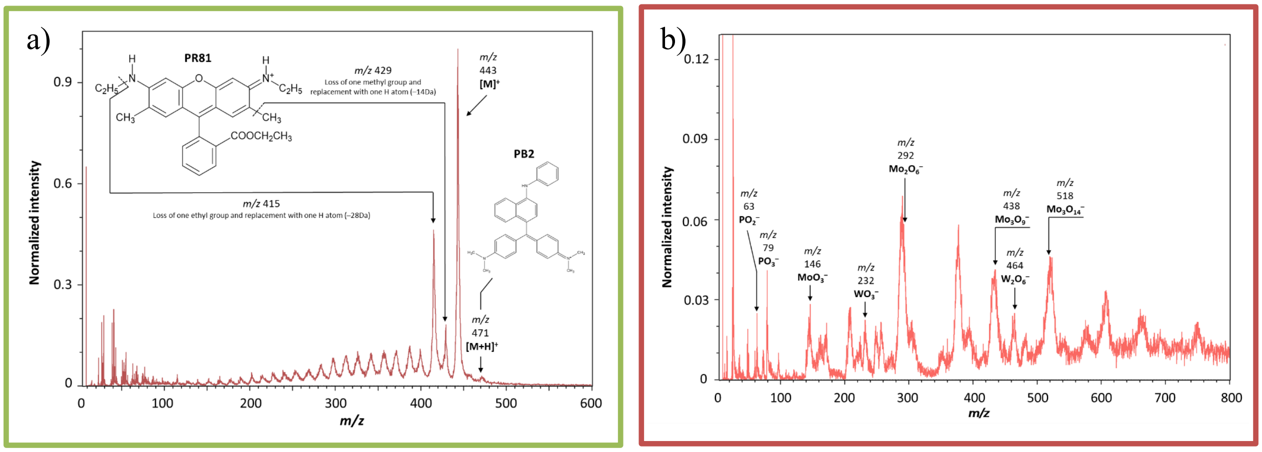

Figure S-15. The positive- (a) and negative-ion (b) modes (5 MeV Si^{4+}) mass spectra of sample 503 (Dragon Purple) – J. S. & W. R. Eakins.

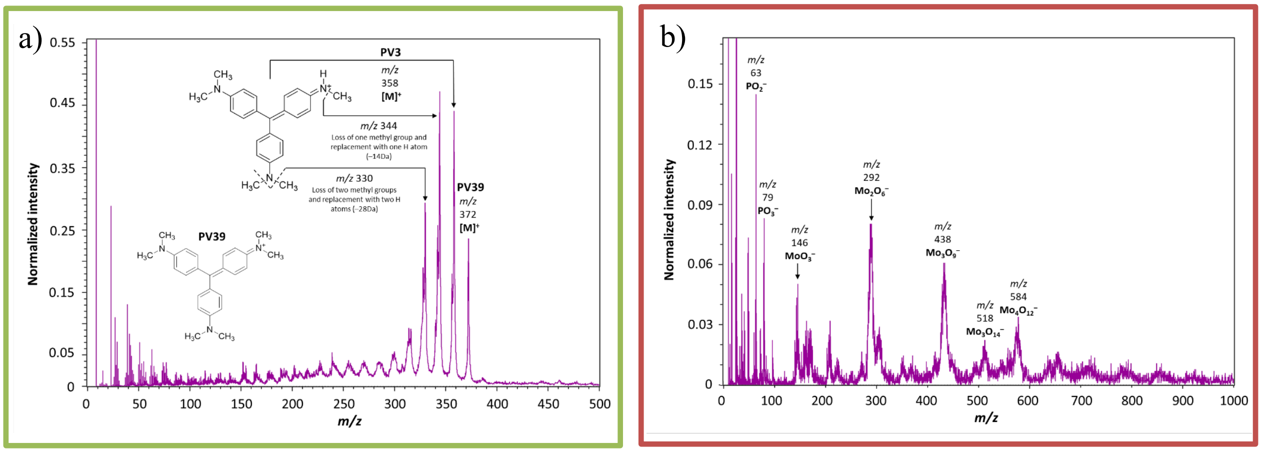

Figure S-16. The positive- (a) and negative-ion (b) modes (5 MeV Si^{4+}) mass spectra of sample 504 (Climatone Blue Toner) – J. S. & W. R. Eakins.

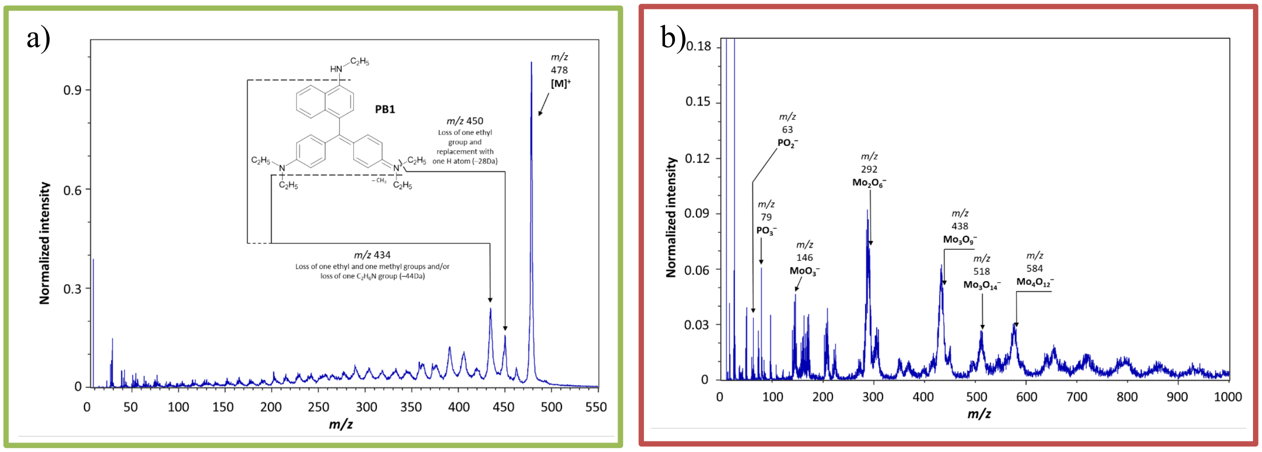

Figure S-17. The positive- (a) and negative-ion (b) modes (5 MeV Si^{4+}) mass spectra of sample 513 (Climatone Purple Toner) – J. S. & W. R. Eakins.

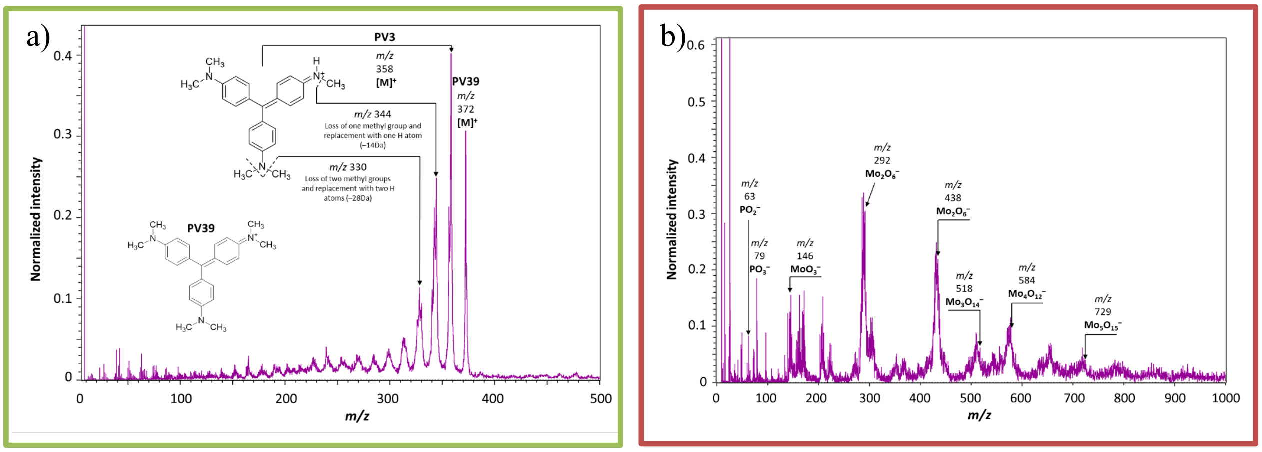

Figure S-18. The positive- (a) and negative-ion (b) modes (5 MeV Si^{4+}) mass spectra of sample 537 (Fastel Yellow Green GA Supra Powder) – I.C.I.

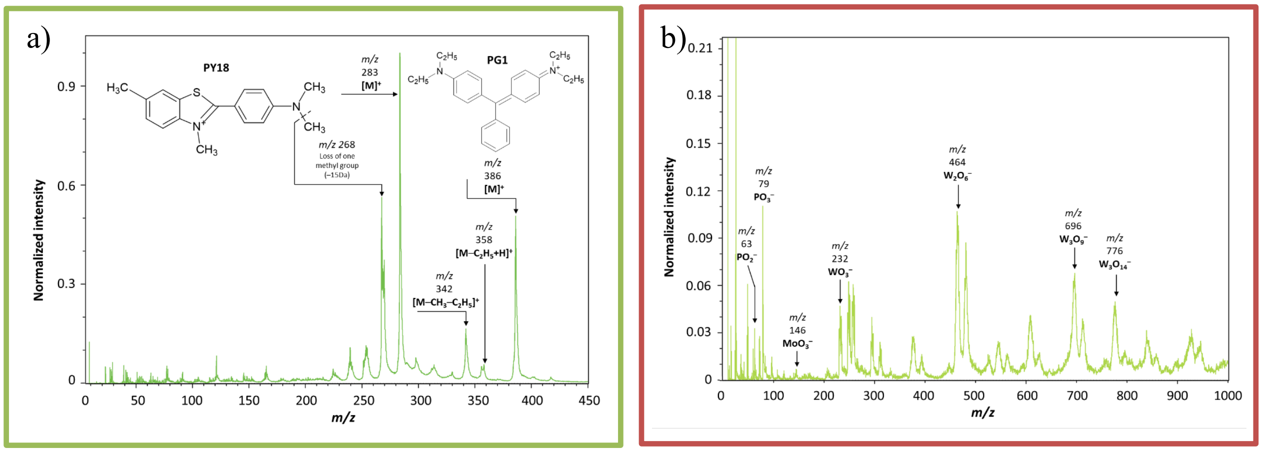

Figure S-19. The positive- (a) and negative-ion (b) modes (5 MeV Si^{4+}) mass spectra of sample 546 (Brillfast Red 6114) – J.W. & T.A. Smith.

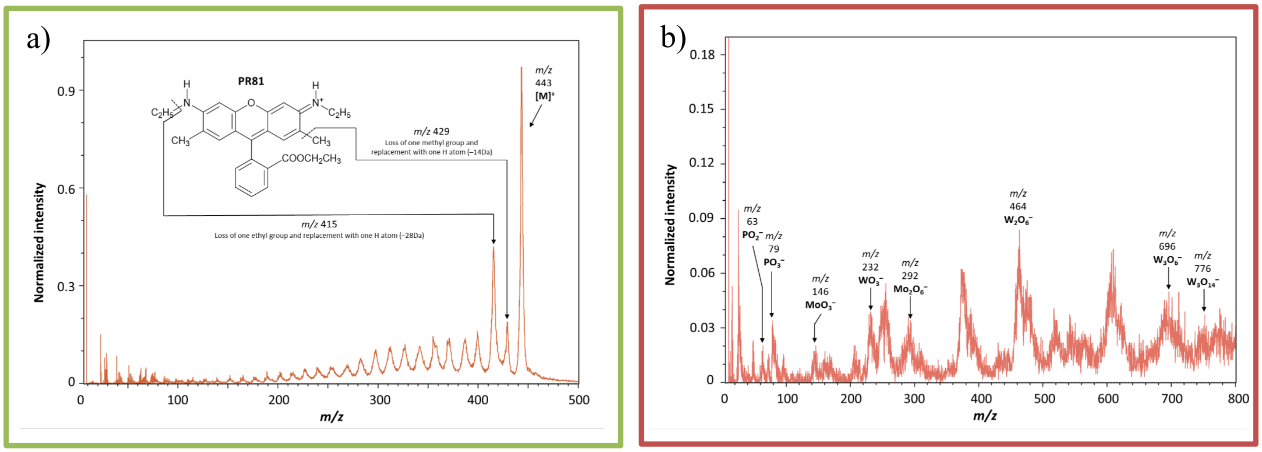

Figure S-20. The positive- (a) and negative-ion (b) modes (5 MeV Si^{4+}) mass spectra of sample 556 (Fastel Blue B Supra Powder) – I.C.I.

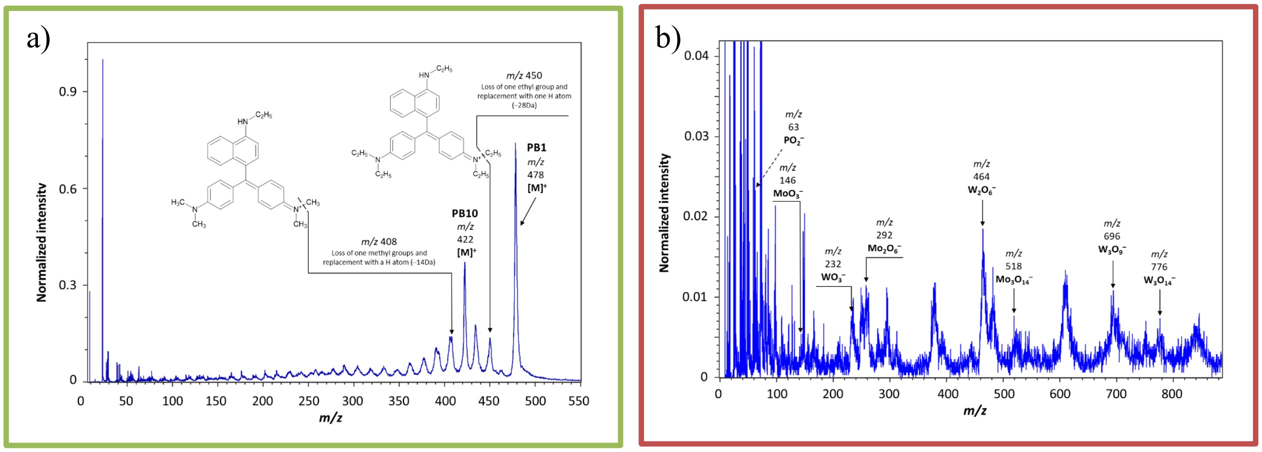

Figure S-21. The positive- (a) and negative-ion (b) modes (5 MeV Si^{4+}) mass spectra of sample 573 (Fastel Violet R Supra Powder) – I.C.I.

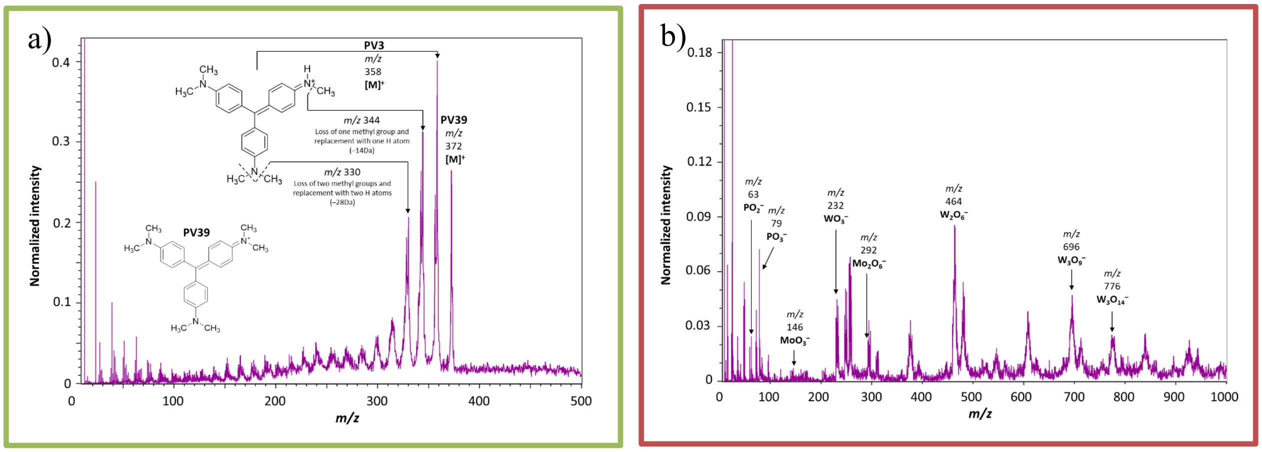

Figure S-22. The positive- (a) and negative-ion (b) modes (5 MeV Si^{4+}) mass spectra of sample 577 (Fastel Pink 2B Supra Powder) – I.C.I.

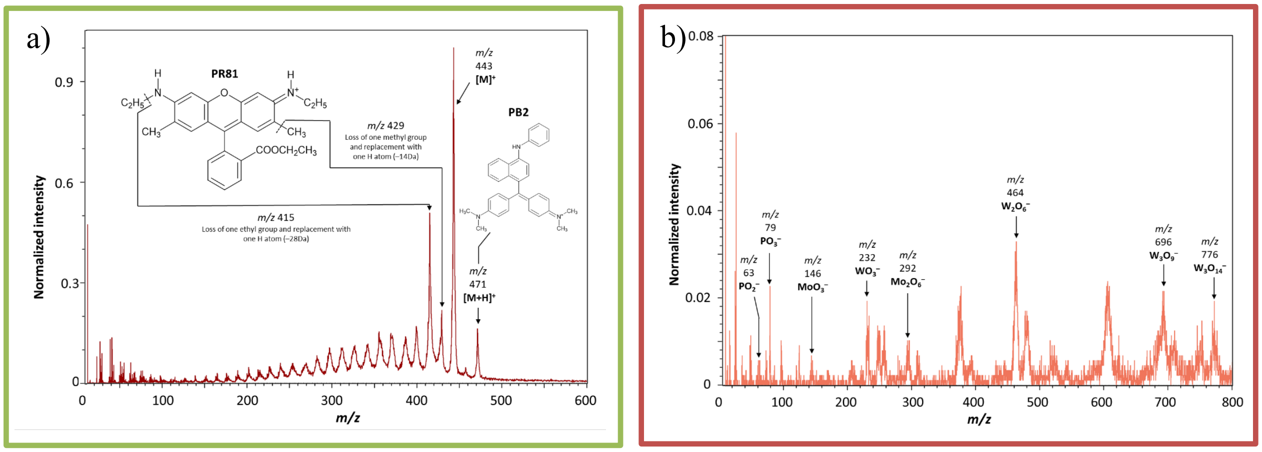

Figure S-23. The positive- (a) and negative-ion (b) modes (5 MeV Si^{4+}) mass spectra of sample 584 (Irgalite azur blue T C R) – Geigy.

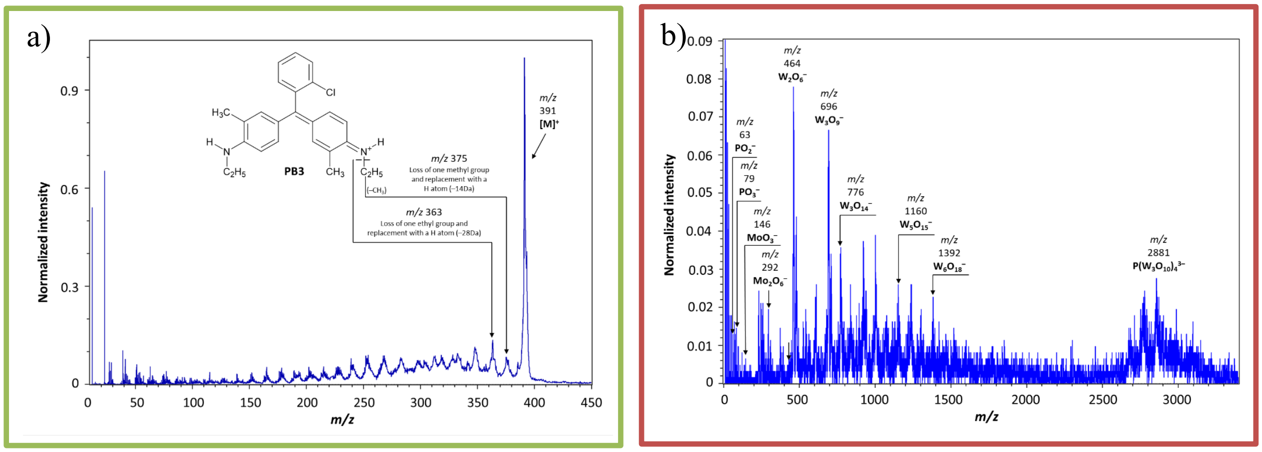

Figure S-24. The positive-ion mode (5 MeV Si^{4+}) mass spectrum of sample 594 (Vert clair Lumière) – Cappelle Frères.

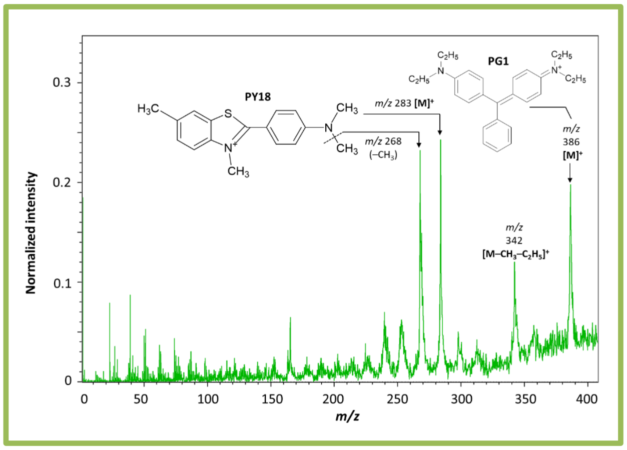

Figure S-25. The positive- (a) and negative-ion (b) modes (5 MeV Si^{4+}) mass spectra of sample 595 (Vert forte Lumière) – Cappelle Frères.

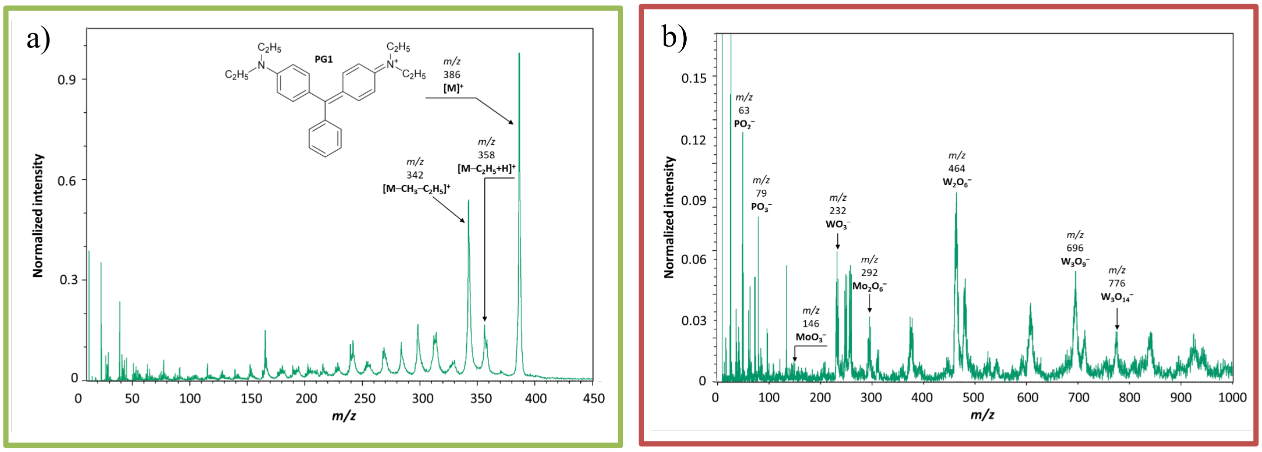

Figure S-26. The positive- (a) and negative-ion (b) modes (5 MeV Si^{4+}) mass spectra of sample 602 (Irgalite Blue T C S) – Geigy.

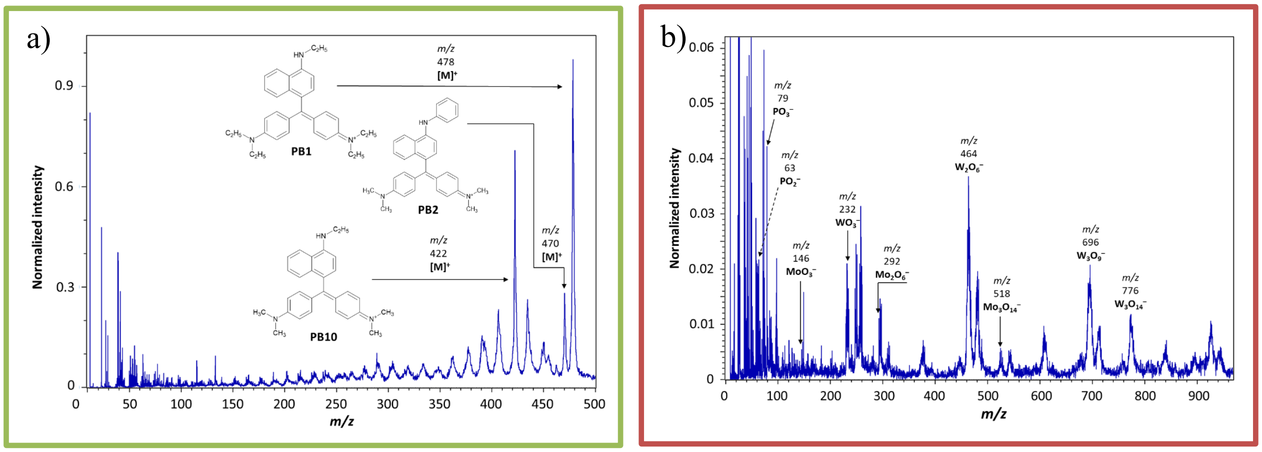

Figure S-27. The positive- (a) and negative-ion (b) modes (5 MeV Si^{4+}) mass spectra of sample 606 (Irgalite Violet TCR) – Geigy.

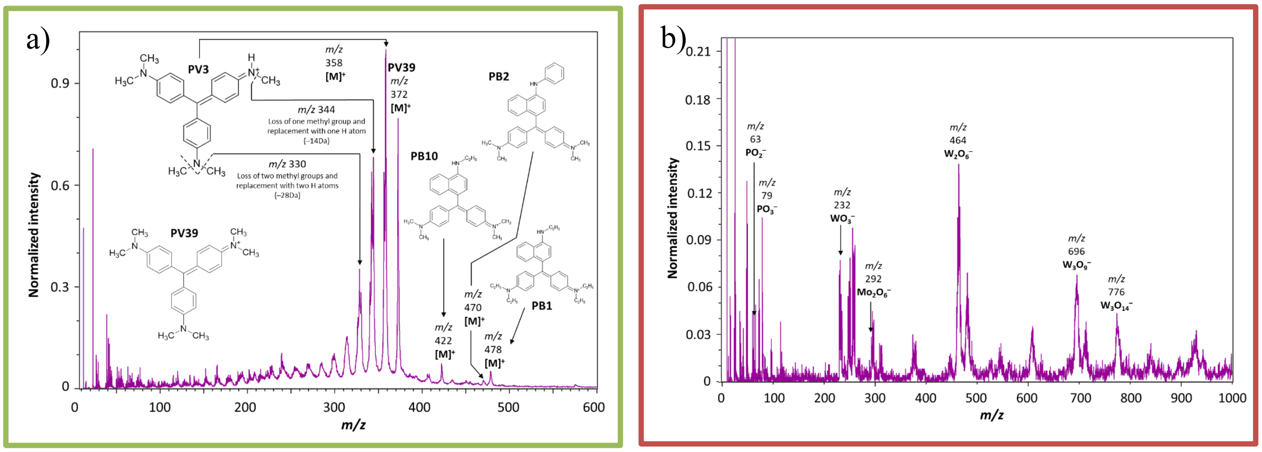

Figure S-28. The positive- (a) and negative-ion (b) modes (5 MeV Si^{4+}) mass spectra of sample 623 (Brillfast Sky Blue 3862) – J.W. & T.A. Smith.

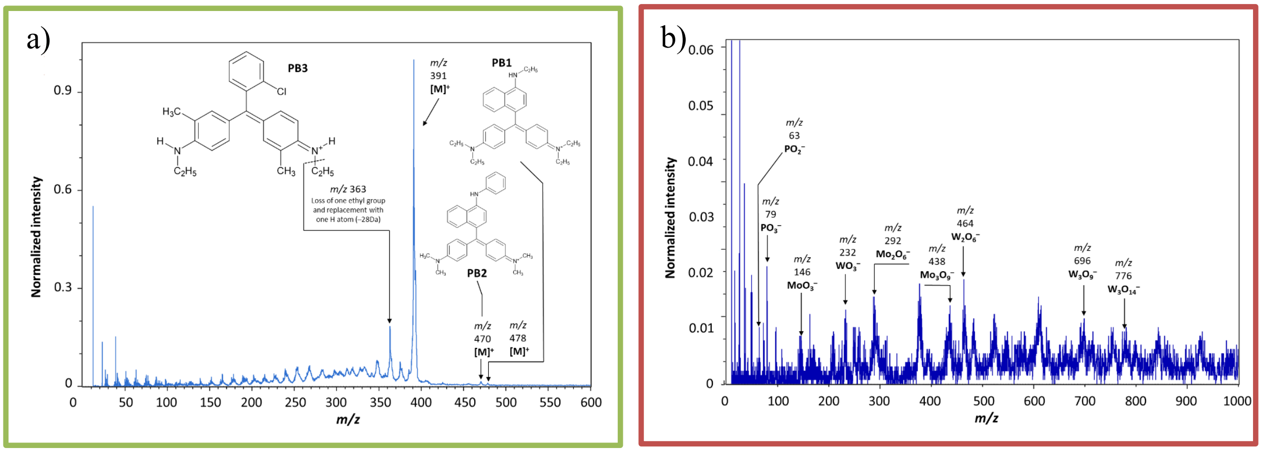

Figure S-29. The positive- (a) and negative-ion (b) modes (5 MeV Si^{4+}) mass spectra of sample 624 (Brillfast Maltese Blue 3591) – J.W. & T.A. Smith.

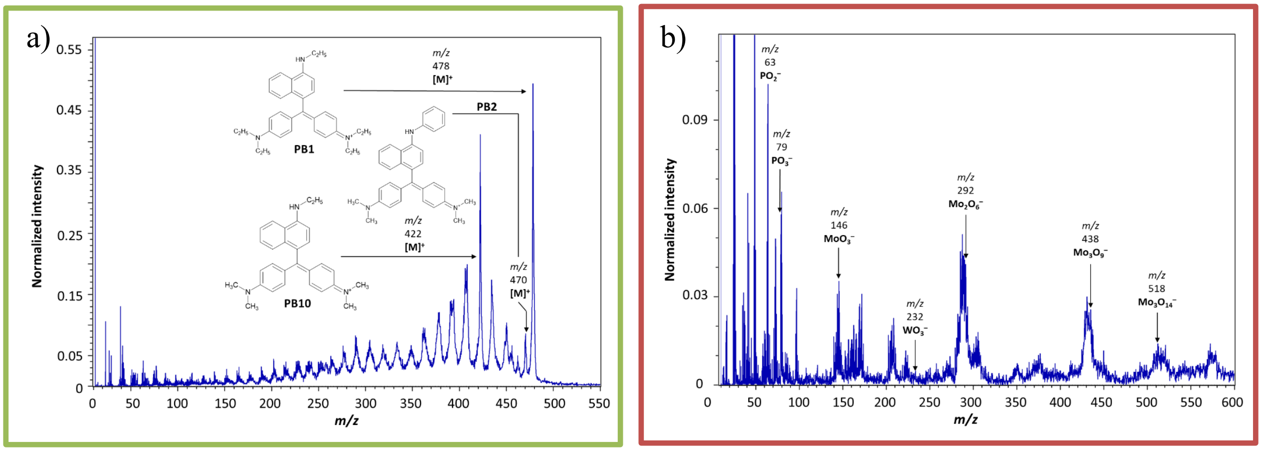

Figure S-30. The positive- (a) and negative-ion (b) modes (5 MeV Si^{4+}) mass spectra of sample 627 (Brillfast Deep Green) – J.W. & T.A. Smith.

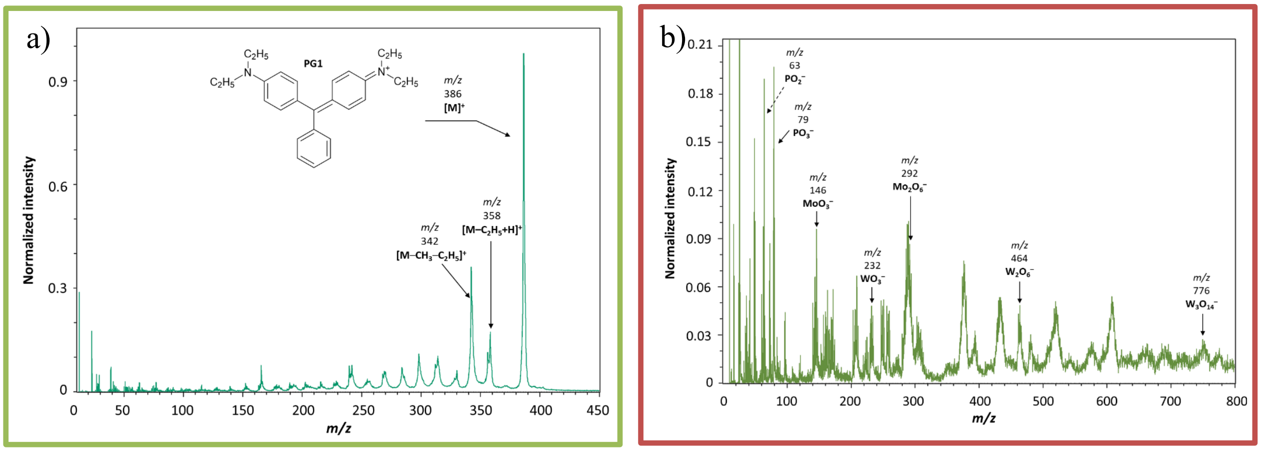

Figure S-31. The positive- (a) and negative-ion (b) modes (5 MeV Si^{4+}) mass spectra of sample 867 (Fastel Pink R Supra Powder) – I.C.I.

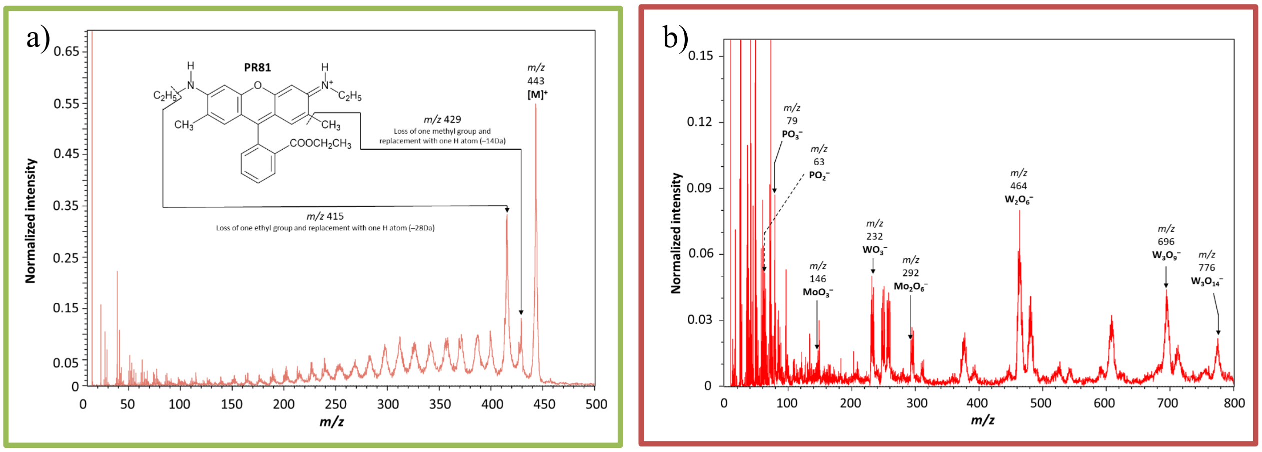

Table S-3 presents the summarized results of the previous investigations with X-ray florescence (XRF), micro-Raman, and Fourier-transform infrared (FTIR) spectroscopies[1] on the same colorant samples from the INTK's collection as analyzed in this work. The following setup and experimental parameters have been used for the analyses:

- XRF analysis was performed with a TRACOR XRAY SPECTRACE5000 (Thermo Electron Corp.) instrument equipped with a Rh tube and a SiLi detector. The tube voltage was set to 8, 30, and 50 kV. When the voltage was set to 8 kV the analysis was conducted in vacuum, while when set to 30 and 50 kV it was performed in air. All three tube voltages were used for XRF spectra acquisition per sample. Measuring time for all spectra was set to 60 s.
- Micro-Raman analysis was performed with the dispersive confocal Raman instrument LabRAM Aramis (Horiba) equipped with three laser sources: either 532 nm (Nd:YAG, 50 mW), 633 nm (HeNe, 17 mW), and 785 nm (Diode, 80 mW). Measuring parameters, especially measuring time laser, and laser power were selected individually in order to obtain the best quality spectra. The acquired spectra were compared to those from the SOPRANO open access library provided by KIK-IRPA.[2]
- FTIR analysis was conducted with a Spectrum 2000 (Perkin Elmer) instrument combined with a microscope (i-Series). The analyses of powder samples were performed with diamond micro-compression cell using the following parameters: 200 scans, 4 cm^{-1} resolution, software internal CO_2/H_2O correction.

Table S-3. Results gained form previous work with XRF, FTIR[1] and micro-Raman (unpublished data), alongside the MeV SIMS results from this work.

samples (INTK inv. no.)	chemical class	chemical elements (ordered by atomic number Z) detected with XRF	Raman bands (bands intensity: vw – very weak, w – weak, m – moderate, s- strong, vs – very strong) The laser used for acquisition of Raman spectra is depicted in **bold**.	XRF, FTIR and micro-Raman results	MeV SIMS identified colorants (in **bold**) and precipitating agents (if present)
140	triarylcarbonium toner	Ca, Fe, Sr, Mo, W	1620s, 1588m, 1533vw, 1478w, 1448vw, 1384s, 1293m, 1178s, 937w, 917m, 810m, 761m, 529m, 438m, 424m, 334w, 222vs, 120w – **633 nm**	**PV3** (PTMA)	**PV3, PV39, PB1**, **PB10** (PTMA)
141	triarylcarbonium toner	Ca, Sr, Mo, Ba, W	1620s, 1587m, 1533vw, 1479w, 1447vw, 1377s, 1297m, 1179s, 937w, 918m, 810m, 762m, 736m, 529m, 440m, 424m, 334w, 222vs – **785 nm**	**PV3** (PTMA)	**PV3, PV39, PV3** (PTMA)
142	triarylcarbonium toner	Al, Si, Ca, V, Fe, Co, Sr, Mo, Ba, W, Pb	1620m, 1590w, 1531vw, 1480w, 1448vw, 1379m, 1298m, 1182s, 937w, 919m, 816m, 737m, 762m, 530m, 443m, 424m, 335w, 220vs, 120w – **633 nm**	**PV3** (PTMA)	**PV3, PV39, PB2, PR81**
144	triarylcarbonium toner	Al, Ca, Fe, Sr, Mo, Ba, W, Pb	1620m, 1592w, 1531vw, 1484w, 1443vw, 1381m, 1300m, 1183s, 919m, 815m, 762m, 735m, 530m, 443m, 422m, 334w, 220vs, 118w – **633 nm**	**PV3** (PTMA)	**PV3, PV39, PB2**
215	β-naphthol pigment	Cl, Ca, Fe	1622m, 1554w, 1497m, 1446s, 1395m, 1333s, 1321s, 1254w, 1217m, 1187vs, 1130m, 986m, 924m, 843m, 794m, 723m, 672m, 617w, 455s, 381m, 339s – **633 nm**	**PR3**	**PR3**

Table S-3. (continued)

samples (INTK inv. no.)	chemical class	chemical elements (ordered by atomic number Z) detected with XRF	Raman bands (bands intensity: vw – very weak, w – weak, m – moderate, s- strong, vs – very strong) The laser used for acquisition of Raman spectra is depicted in **bold**.	XRF, FTIR and micro-Raman results	MeV SIMS identified colorants (in **bold**) and precipitating agents (if present)
226	β-naphthol pigment	S, Ca, Ba	1624vw, 1609w, 1590m, 1552w, 1493w, 1481vw, 1454w, 1429w, 1395m, 1369w, 1340m(sh), 1329vs, 1292vw, 1256w, 1250w, 1223w, 1185w, 1167w, 1156w, 1140vw, 1105s, 1000w, 985m, 873w, 860m, 752vw, 726w, 632w, 614w, 560w, 534vw, 460w, 425w, 407w, 360w, 324w, 268w, 183s, 121m – **785 nm**	**PR1** or **PR4**	**PR1**, **PR3**
231	β-naphthol pigment	Cl, Ca, Fe, Sr, Pb	1620m, 1605w, 1562vw, 1553w, 1525vw, 1495m, 1480vw, 1465vw, 1444m, 1395m, 1332s, 1319m, 1307w, 1254w, 1248w, 1223w, 1214m, 1184m, 1157w, 1127m, 1097vw, 1081w, 1076w, 1039vw, 984m, 924w, 842w, 795m, 744vw, 722m, 676vw, 646vw, 616m, 542vw, 502w, 478w, 454m, 421vw, 402vw, 381m, 363vw, 339s, 252w, 195w, 165w, 137w, 122w – **785 nm**	**PR3**	**PR3**
232	β-naphthol lake	S, Ca	1617m, 1603m, 1559w, 1552w, 1481s, 1465s, 1449m, 1425s, 1413s, 1350s, 1323vw, 1257w, 1234w, 1216s, 1200vs, 1171vw, 1154w, 1147w, 1141w, 1134w, 1096m, 1059vw, 1034vw, 990w, 952vw, 920vw, 825vw, 799vw, 775vw, 719vs, 662vw, 644w, 605m, 544w, 527m, 481w, 468m, 433vw, 422vw, 410m, 376vw, 358w, 342s, 300m, 225w, 200m, 146w, 110s – **785 nm**	**PR49**	**PR49**
248	β-naphthol lake	S, Cl, Ca, Ba	1618vw, 1603w, 1596w, 1571w, 1553m, 1481m, 1451w, 1400vs, 1377vw, 1333m, 1317w, 1291vw, 1259w, 1236s, 1198m, 1159vw, 1139vw, 1095m, 1039vw, 994vw, 978w, 871vw, 805w, 740vw, 720m, 667vw, 631m, 612m, 578vw, 557vw, 540vw, 515w, 497w, 435m, 426m, 353vw, 338m, 310m, 284vw, 243vw, 203w, 171w, 115m(sh) – **785 nm**	**PR53:1**	**PR53:1**
250	β-naphthol pigment	S, Cl, Ca	1620m, 1605w, 1562vw, 1553w, 1525vw, 1495m, 1480vw, 1467vw, 1444m, 1395m, 1332s, 1319m, 1307w, 1254w, 1249w, 1222w, 1215m, 1185m, 1158w, 1128m, 1098vw, 1081w, 1076w, 1040vw, 984m, 924w, 842w, 796m, 761vw, 744vw, 722m, 676vw, 646vw, 616m, 542vw, 533vw, 502w, 478w, 454m, 421vw, 401vw, 381m, 364vw, 339s, 252w, 195m, 165m, 137m, 120m – **785 nm**	**PR3**	**PR3**
411	triarylcarbonium toner	Si, S, Sr, Mo, Ba, W	1614vs, 1591m, 1533vw, 1490w, 1450vw, 1426w, 1379m, 1366m, 1291m, 1216m, 1181s, 1161m, 1076w, 916m, 807w, 750w, 734w, 699vw(sh), 437m, 412w, 259m(sh), 230s, 117w – **633 nm**	**PG1** (PTMA)	**PG1** (PTMA)
450	triarylcarbonium toner	Si, S, Ca, Sr, Mo, Ba, W	1615vs, 1592s, 1534vw, 1490w, 1449vw, 1425m, 1379m, 1365s, 1290m, 1217m, 1182s, 1160m, 1076w, 916m, 804m, 750w, 735w, 699vw, 531w, 439m, 415m, 259m(sh), 230s, 117w – **633 nm**	**PG1**, **PG2**, or **PG4** (PTMA)	**PG2** (PG1 + PY18) (PTMA)
464	triarylcarbonium toner	Al, Si, Ca, Cr, Sr, Mo, Ba, W, Pb	1626vs, 1599s, 1547vw, 1503m, 1461w, 1437w, 1393m, 1373m, 1346m, 1299m, 1226m, 1186m, 1167w, 1150m, 1078w, 921m, 809m, 752w, 705vw, 531vw, 438m, 421m, 354vw, 266w(sh), 236w, 106w – **532 nm**	**PG1**, **PG2**, or **PG4** (PTMA)	**PG2** (?) (PG1 + a diarylide yellow) (PTMA)
489	triarylcarbonium toner	Si, Ca, Mo, W	1645m, 1598vw, 1577w, 1506s, 1363s, 1189m, 1129vw, 776m, 639vw, 615s, 404vw – **633 nm**	**PR81** (PTMA)	**PR81**, **PB2** (PTMA)
503	triarylcarbonium toner	Al, Si, Fe, Sr, Mo, Ba	1619m, 1586w, 1481w, 1442vw, 1378m, 1299m, 1179s, 917m, 811m, 761m, 734m, 530m, 441m, 423m, 334w, 219vs, 119w – **633 nm**	**PV3** (PMA)	**PV3, PV39** (PMA)
504	triarylcarbonium toner	Ca, Fe, Mo, Pb	1615s, 1568m(sh), 1389s, 1298w, 1202s(sh), 1177s, 1073vw, 918w, 863vw, 761w, 684w, 659w, 471w, 434w, 224vs – **633 nm**	**PB1** (PMA)	**PB1** (PMA)

Table S-3. (continued)

samples (INTK inv. no.)	chemical class	chemical elements (ordered by atomic number Z) detected with XRF	Raman bands (bands intensity: vw – very weak, w – weak, m – moderate, s- strong, vs – very strong) The laser used for acquisition of Raman spectra is depicted in **bold**.	XRF, FTIR and micro-Raman results	MeV SIMS identified colorants (in **bold**) and precipitating agents (if present)
513	triarylcarbonium toner	Ca, Fe, Mo	1618m, 1588m, 1529vw, 1477w, 1447vw, 1376s, 1298m, 1180s, 918m, 810m, 762w, 732vw, 529m, 437m, 419m, 336w, 226vs – **633 nm**	**PV3** (PMA)	**PV3, PV39** (PMA)
537	triarylcarbonium toner	S, Fe, Mo, W	1617vs, 1592s, 1490w, 1451vw, 1428m, 1384m, 1365s, 1296s, 1282m, 1220m, 1184s, 1159m, 1076w, 916m, 803m, 750w, 733w, 697vw, 531w, 441m, 415m, 222s, 114w – **633 nm**	**PG2** (PTMA)	**PG2** (PG1 + PY18) (PTMA)
546	triarylcarbonium toner	Si, S, Mo, Ba, W,	1647m, 1595vw, 1573w, 1507s, 1361s, 1308m, 1184m, 1129w, 773m, 639vw, 613m, 398w, 272w – **633 nm**	**PR81** (PTMA)	**PR81** (PTMA)
556	triarylcarbonium toner	Mo, W	1615s, 1568m(sh), 1389s, 1298w, 1202s(sh), 1177s, 1073vw, 918w, 863vw, 761w, 684w, 659w, 471w, 434w, 224vs – **633 nm**	**PB1** (PTMA)	**PB1**, **PB10** (PTMA)
573	triarylcarbonium toner	Si, Ca, V, Mo, W	1616m, 1589m, 1528vw, 1477w, 1439vw, 1377m, 1298m, 1180s, 918m, 811m, 759m, 733w, 528m, 438m, 415m, 333vw, 222m, 119w – **633 nm**	**PV3** (PTMA)	**PV3, PV39** (PTMA)
577	triarylcarbonium toner	Si, S, Ca, Mo, W	1643m, 1594vw, 1573w, 1503s, 1356s, 1305m, 1181m, 1120w, 769m, 637vw, 609m, 519w, 394w, 272w – **633 nm**	**PR81** (PTMA)	**PR81**, **PB2** (PTMA)
584	triarylcarbonium toner	Si, Cl, Ca, Mo, W	1625s, 1589m, 1401w, 1318m, 1264w(sh), 1155m, 1117w, 1025vw, 948w, 832m, 760m, 685m, 576m, 262s, 225vs – **633 nm**	**PB3** (PTMA)	**PB3** (PTMA)
594	triarylcarbonium toner	Si, S, Cl, Ca, Mo, Ba, W	1618vs, 1594m, 1491m, 1451w, 1430m, 1384m(sh), 1366s, 1296m, 1284m, 1221m, 1185s, 1160m, 1080w, 917w, 906w(sh), 803w, 752w, 734w, 700vw, 535vw, 463m(sh), 443m, 267vw, 223s, 116vw – **633 nm**	**PG1, PG2,** or **PG4** (PTMA)	**PG2** (PG1 + PY18) (PTMA)
595	triarylcarbonium toner	Cl, Ca, Mo, W	1618vs, 1592w, 1534vw, 1490w, 1429m, 1383m, 1366m, 1296m, 1284m, 1221m, 1185m, 1161m, 1077w, 918w, 802m, 752w, 735w, 699vw, 442m, 265w(sh), 222m, 117w – **633 nm**	**PG1** (PTMA)	**PG1** (PTMA)
602	triarylcarbonium toner	Si, Ca, Mo, W	1610s, 1565m(sh),1388m, 1298m, 1153m, 1204s, 1179s, 1072w, 918m, 860w, 761m, 735w, 681vw, 684m, 660w, 471w, 219vs – **633 nm**	**PB1** (PTMA)	**PB1**, **PB2**, **PB10** (PTMA)
606	triarylcarbonium toner	Al, Si, Cl, Ca, V, Mo, W	1618s, 1586w, 1531vw, 1479w, 1442vw, 1378m, 1298m, 1177s, 916m, 810m, 759m, 732w, 527m, 441m, 423m(sh), 332w, 216vs, 117m – **633 nm**	**PV3** (PTMA)	**PV3**, PV39, **PB1**, **PB2**, **PB10** (PTA)
623	triarylcarbonium toner	Si, S, Cl, Mo, Ba, W	1623s, 1588w, 1400w, 1318m, 1271m, 1260w(sh), 1153m, 1117w, 1024vw, 833m, 757w, 684w, 574m, 262s – **633 nm**	**PB3** (PTMA)	**PB3**, **PB1**, **PB2** (PTMA)
624	triarylcarbonium toner	S, Mo, Ba, W	1611s, 1569m(sh), 1361m, 1259vw, 1153m, 1199m(sh), 1178s, 1071vw, 916w, 863vw, 757vw, 681vw, 656vw, 471vw, 219m – **633 nm**	**PB1** (PTMA)	**PB1**, **PB2**, **PB10** (PTMA)
627	triarylcarbonium toner	Si, S, Mo, Ba, W	1616vs, 1593w, 1534vw, 1490w, 1451vw, 1427w, 1384w, 1365m, 1296m, 1294m, 1219w, 1184m, 1159w, 1076vw, 917w, 803w, 752vw, 735vw, 699vw, 440m, 262w(sh), 228s, 117w – **633 nm**	**PG1** (PTMA)	**PG1** (PTMA)
867	triarylcarbonium toner	Si, Ca, Mo, W	1650m, 1597vw, 1576w, 1508s, 1364s, 1310m, 1186m, 1129w, 1092vw, 775m, 662vw, 639vw, 614s, 522w, 462vw, 395w, 308w, 273w, 165w – **633 nm**	**PR81** (PTMA)	**PR81** (PTMA)

REFERENCES

(1) Schäning, A. Synthetische organische Farbmittel aus einer technologischen Materialsammlung des 19./20. Jahrhunderts: Identifizierung, Klassifizierung und ihre Verwendung sowie Akzeptanz in (Künstler)Farben Anfang des 20. Jahrhunderts. Dissertation, Akademie der bildenden Künste Wien, Wien, 2010.

(2) Fremout, W.; Saverwyns, S. Identification of Synthetic Organic Pigments: The Role of a Comprehensive Digital Raman Spectral Library. J. Raman Spectrosc. 2012, 43 (11), 1536–1544, DOI: 10.1002/jrs.4054
